# Removal of hydrogen sulfide from a biogas mimic by using impregnated activated carbon adsorbent

**DOI:** 10.1371/journal.pone.0211713

**Published:** 2019-02-12

**Authors:** Nurul Noramelya Zulkefli, Mohd Shahbudin Masdar, Wan Nor Roslam Wan Isahak, Jamaliah Md Jahim, Syahril Anuar Md Rejab, Chew Chien Lye

**Affiliations:** 1 Research Centre for Sustainable Process Technology (CESPRO), Faculty of Engineering and Built Environment, Universiti Kebangsaan Malaysia, UKM Bangi, Selangor, Malaysia; 2 Program of Chemical Engineering, Faculty of Engineering and Built Environment, Universiti Kebangsaan Malaysia, UKM Bangi, Selangor, Malaysia; 3 Fuel Cell Institute, Universiti Kebangsaan Malaysia, UKM Bangi, Selangor, Malaysia; 4 Sime Darby Research Sdn. Bhd., Jalan Pulau Carey, Pulau Carey, Selangor, Malaysia; King Saud University, SAUDI ARABIA

## Abstract

Adsorption technology has led to the development of promising techniques to purify biogas, i.e., biomethane or biohydrogen. Such techniques mainly depend on the adsorbent ability and operating parameters. This research focused on adsorption technology for upgrading biogas technique by developing a novel adsorbent. The commercial coconut shell activated carbon (CAC) and two types of gases (H_2_S/N_2_ and H_2_S/N_2_/CO_2_) were used. CAC was modified by copper sulfate (CuSO_4_), zinc acetate (ZnAc_2_), potassium hydroxide (KOH), potassium iodide (KI), and sodium carbonate (Na_2_CO_3_) on their surface to increase the selectivity of H_2_S removal. Commercial H_2_S adsorbents were soaked in 7 wt.% of impregnated solution for 30 min before drying at 120°C for 24 h. The synthesized adsorbent’s physical and chemical properties, including surface morphology, porosity, and structures, were characterized by SEM-EDX, FTIR, XRD, TGA, and BET analyses. For real applications, the modified adsorbents were used in a real-time 0.85 L single-column adsorber unit. The operating parameters for the H_2_S adsorption in the adsorber unit varied in L/D ratio (0.5–2.5) and feed flow rate (1.5–5.5 L/min) where, also equivalent with a gas hourly space velocity, GHSV (212.4–780.0 hour^-1^) used. The performances of H_2_S adsorption were then compared with those of the best adsorbent that can be used for further investigation. Characterization results revealed that the impregnated solution homogeneously covered the adsorbent surface, morphology, and properties (i.e., crystallinity and surface area). BET analysis further shows that the modified adsorbents surface area decreased by up to 96%. Hence, ZnAc_2_–CAC clarify as the best adsorption capacity ranging within 1.3–1.7 mg H_2_S/g, whereby the studied extended to adsorption-desorption cycle.

## Introduction

The increment of global energy demand is due to the continuous population growth, and the economy steeping up, which affected the socio-economic landscape and human welfare in the future [1]. The renewable energy and fossil fuel are incorporated as future energy systems that control and conserve the fossil fuel used which had been discovered for future continuity demands. Hence, the sources of alternative renewable energy from the biomass resources had found as relevant continuous energy supplies based on the constant based load, control of resources and production of resources.

Biogas is formed from a gas mixture through degradation of organic matter that caused by several microorganisms under anaerobic condition. Table 1 shows the gas composition of biogas from various biogas resources [2–6]. Biogas has the most rigorous quality specification when intended to be used as a natural gas substitute. For instance, the draft of the European regulation on biomethane is targeting a composition of CH_4_ > 95%, CO_2_ < 2.5–4%, O_2_ < 0.001–1%, H_2_S + COS < 5 mg Nm^−3^, NH_3_ < 10 mg Nm^−3^, BTX < 500 mg Nm^−3^, and siloxanes < 10 mg Nm^−3^ [7]. However, the concentration of H_2_S gas which is found to range between as low as about 50–10,000 ppm depending on the feed material composition to the digester [8].

**Table 1:** The composition of several components in biogas production [2].

Components	Composition, %
CO_2_	30–40
CH_4_	60–70
H_2_S	0.15–3.0
NH_3_	< 1
N_2_	0–2
CO	< 0.6
O_2_	0–1
H_2_O	5–10

As in the application for fuel cell technology development, such as Polymer Electrolyte Membrane Fuel Cell (PEMFC), and Solid Oxide Fuel Cell (SOFC), the devices commonly use methane and hydrogen as energy supplies. As the biogas might contain unwanted gases such as H_2_S and carbon monoxide (CO), hence, some studied recommended the tolerable concentration range should be <1 ppm and <10 ppm [9–15]. The tolerable concentration of H_2_S gas for fuel cell devices are important to follow as H_2_S gas are known as a toxic gas. Besides, other researchers recommended to remove at the early stage of purification due to the several possibilities that may be affected to the quality of biomethane production, causes corrosion on mechanical wear, and emits harmful substances to the fuel cell catalyst.

Thus, the elimination of H_2_S and CO_2_ gas undergoes by several technologies, such as biological [16], chemical absorption (scrubbing with active liquids) [17], adsorption by using mesoporous material [9], and membrane-based gas permeation technologies [18]. The biological oxidation method, absorption, and membrane-based gas permeation are less efficient due to having a higher cost, and limited desulphurization efficiency [18]. By contrast, the adsorption technologies are efficient and preferable for the low concentration of H_2_S removal in biogas system [19–22].

Activated carbon is commonly used in adsorption due to its high surface area, microporosity, thermal stability, high removal capacity, and low cost per unit volume of adsorber compared with the other mesoporous material, such as zeolite, metal-organic, porous silica, and clay incandescent. In addition, its surface property, i.e., pore volume, surface area, and chemistry, determines the overall adsorption performance, and its capability in pollutant treatment was proven by the previous study on Nitrogen Oxide (NO), H_2_S, and volatile organic compounds [20–23].

Researchers discovered that impregnated activated carbon modifies and enhances adsorption performance by increase the number of active sites on the adsorbents surface [24]; however, results differ for different gases [25]. Hence, the materials used for H_2_S adsorption had appropriate physico-chemical properties; the superficial properties in terms of specific surface, size, and distribution of pores. The H_2_S gas compounds are very small; thus, adsorbents with well-developed microporosity were used [26]. Selection of appropriate material for adsorption is important to prevent secondary waste stream problems since activated carbon is non-regenerable product [27].

Impregnated activated carbon for H_2_S adsorbents, which used alkaline compounds, such as sodium hydroxide (NaOH), potassium hydroxide (KOH), and Potassium Carbonate (K_2_CO_3_), for adsorbent modification was studied by Choo et al. [28]. Furthermore, a study by Sitthikhankaew et al. [29] also used alkaline compounds, such as potassium iodide (KI), Sodium Carbonate (Na_2_CO_3_), KOH, and NaOH. An investigation by Phooratsamee et al. [30] used zinc chloride, K_2_CO_3_, NaOH, and KI as the impregnated compound.

As in this study to use the commercial mixed gas of H_2_S/N_2_/CO_2_, the chosen adsorbents should be capable of capturing both gases (H_2_S and CO_2_). Previously, we studied on the CO_2_ removal from biohydrogen production using this technique of impregnated activated carbon through ionic liquid [31], Na_2_CO_3_, ZnAc_2_ and copper sulfate (CuSO_4_) [32]. Based on these studies, it was confirmed that the impregnation of carboneous material, i.e., activated carbon, could enhance the adsorbent capacity in capturing or removing the unwanted gases such as CO_2_. For instance, the ionic liquid at 1 wt.% [33] and AC-ZnAc_2_ at 7 wt.% [32] show the best adsorbents for CO_2_ adsorption capacity with 84.89 mg/g [31] and 63.61 mg/g compared to that activated carbon without impregnation at 30.44 mg/g [32]. Moreover, in the industrial practice, the ionic liquid could be considered for replacements of conventional organic solvents due to their negligible vapour pressures, high thermal stability and virtually limitless chemical tunability [33]. The activated carbon normally had undergone frequent changes which led to secondary waste. Then, the idea of regeneration, disposed or reused in industries had been reviewed [34].

The regeneration method is likely used as more economic compared to adsorbents replacement, reduced the secondary waste and environmentally friendly [35]. Basically, the carbon regeneration can be divided into four major mechanisms groups which are thermal regeneration, chemical regeneration, microbiological regeneration, and vacuum regeneration [36]. However, the thermal regeneration practically used in the industries [37].

In this study, 7 wt.% of the impregnated chemical over activated carbon had known as adsorbent were synthesized for removal of unwanted H_2_S gas using adsorption technique. The characterization of these adsorbents such as surface morphology, porosity, and structures, were determined using the SEM-EDX, FTIR, XRD, TGA, and BET analysis. For real adsorption approach, different types of commercial mixed gas (H_2_S/N_2_ and H_2_S/N_2_/CO_2_) were used to investigate the performance and efficiency of H_2_S adsorption by manipulating the operating parameters such as gas flow rate, adsorbents types, and L/D (Length per diameter) ratio. The selection of H_2_S concentration range used in this study was higher compared to the actual concentration found in biomethane production because the focus of this study literally not only on H_2_S production from biomethane but also could be used in others H_2_S production from difference feed composition. In fact, the difference feed composition resulted for difference range of H_2_S concentration.

Based on the results, the ZnAc_2_-CAC shows a higher capability in capturing the H_2_S gas had led the study of adsorption-desorption cycle by using a higher concentration of commercial mixed gas H_2_S/N_2_ for economical purposed. Whereas, the operating parameter used are different with other researchers which normally operated at a minimal value of operating parameters such as flow rate and amount of adsorbent used. Hence, the H_2_S adsorption-desorption performance was discussed based on operation parameters and adsorbent characterization.

## Materials and methods

### Adsorbent materials

The granular CAC (2–36 mm) was supplied by Effigen Carbon Sdn. Bhd, Malaysia. The compounds for impregnation, such as potassium hydroxide (KOH), potassium Iodide (KI), sodium carbonate (Na_2_CO_3_), zinc acetate (ZnAC_2_) and Copper Sulfate (CuSO_4_) were purchased from Friendemann Schmidt Chemicals (Malaysia).

### Preparation of impregnated CAC

Sitthikhankaew et al. proposed that the best adsorbent performance was at 7 weight percentage (wt.%) of the impregnated chemicals per 0.35 kg of activated carbon [29,30], which was valid for certain impregnation materials only [29]. In this study, other materials, referred with constant impregnation ratio, were used to determine the best adsorbents. CAC was soaked for 30 min in 40.8 g/L of impregnated solution, and then dried overnight at 120°C. Activated carbon impregnated by KOH, KI, Na_2_CO_3_, ZnAC_2_, and CuSO_4_ is referred to as KOH–CAC, KI–CAC, Na_2_CO_3_–CAC, ZnAC_2_–CAC, and CuSO_4_–CAC, respectively. Non-impregnated CAC is known as raw CAC.

### Characterization of adsorbents

N_2_ adsorption-desorption measurement at 22°C has carried out with micrometrics ASAP 2020 version 4.02. Before the analysis, the adsorbents sample automatic degasses and evacuate at 5.0 mmHg/s to 300 μmHg and hold for 0 minutes. Then, the specific surface area of the adsorbents sample was determined by application of Brunauer–Emmett–Teller (BET) method, while, the micropore volume was calculated using the t-plot method. Next, the crystallographic structure, chemical composition, and physical properties of the materials were determined using X-ray diffraction (XRD) through the Bruker AXS D8 Advance using Cu Kα (λ = 0.154 nm) radiation.

All the adsorbents sample then undergo the analysis for functional group properties through Fourier Transform Infrared (FTIR) spectrum from Perkin Elmer. The adsorbents sample scanned in the range of 600–4000 cm^-1^. The stability and degradation of samples as a function of temperature were observed using thermal gravimetric analyser (TGA-50) of Shimadzu at 20 ml/min, temperature range: 25–600°C, sample mass: 1 g. The physical and chemical properties of all the adsorbents sample were characterized and investigated to analyse the surface morphology, and elements present by scanning electron microscope (SEM-EDX). The analysis was conducted using FESEM (Merlin Compact) to visualize the crystal shape, structure of adsorbent’s particles, porosity, and elements of samples. The characterization morphology of adsorbents used the accelerating voltage at 3 kV; where the adsorbents dried up for 24 hours at 110°C under the vacuum before undergoes the SEM analysis.

### H_2_S adsorber operation

**Fig 1:**
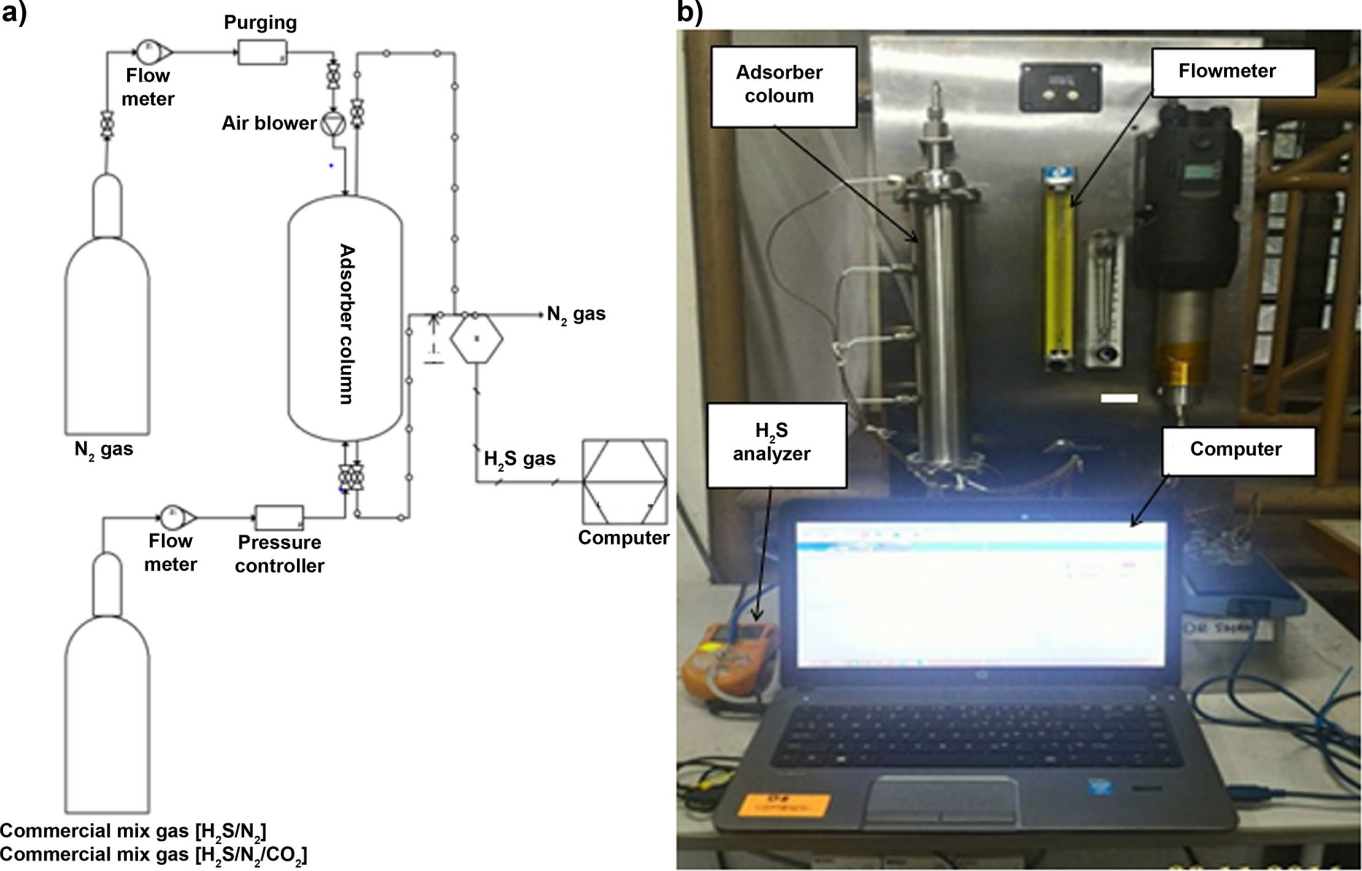
H_2_S adsorption system used in this study. (a) Schematic diagram. (b) Actual photo.

The H_2_S adsorption performances of six adsorbents were examined in a laboratory-scale single adsorber column unit had designed at 0.3 m length. The height of each adsorbent referred to the different adsorbent loads with 0.032 kg, 0.105 kg and 0.155 kg. The adsorption performance was observed by single feeding the commercial mixed gases which provided by Linde Malaysia either for H_2_S/N_2_ (1000 ppm H_2_S balance N_2_) or H_2_S/N_2_/CO_2_ (1000 ppm H_2_S with 49.5 vol.% N_2_ and balanced CO_2_) at ambient temperature. The pressure at the inlet stream was fitted at 1 bar (gauge) for each adsorbent type used. However, the flow rate varied from 1.5–5.5 L/min (GHSV are equivalent to 212.4 hour^-1^ to 780 hour^-1^) and L/D ratio started from 0.5 to 2.5, respectively. Portable H_2_S analyser model GC310 (China) was used to measure H_2_S gas concentration change during the adsorption process. Fig 1(A) and Fig 1(B) shows the schematic diagram and real photo, respectively, for the H_2_S adsorption system.

### Adsorption-desorption cycle

The adsorbent used was ZnAc_2_-CAC, whereas feeding the commercial mixed gases of H_2_S/N_2_ (5000 ppm H_2_S balance N_2_). In purging, three main processes were involved for removal of the excess H_2_S element on the surface adsorbents. During the first 30 min, 100 L/min (14150.9 hour^-1^) of air from the blower entered the column at 150°C. In the next 30 min, another 100 L/min of air fed into the column, without applying any heating process. Finally, for another 30 minutes, the 5.5 L/min of N_2_ gas fed into the column in order to purged out and stabilized the surface adsorbents before ready to use for the next adsorption operation.

### Calculations

#### Adsorption capacity

The adsorption capacity of activated carbon was measured using the breakthrough time, flow rate, and length of bed used, as shown in the following Eq (1) [25]:

$$Q=\frac{q\times T_{B}\times C\times {MW}_{H2S}}{V_{M}\times m_{ads}}$$ (1)

where *Q* (mg H_2_S/g) refers to adsorption capacity, *q* (L/min) is feed flow rate, *T*_*B*_ (min) is breakthrough time, *C* (kg/L) is breakthrough concentration, *MW*_*H2S*_ (kg/kmol) is the molecular weight of H_2_S, *V*_*M*_ (L) is molar volume at S.T.P., and *m*_*ads*_ (kg) is mass adsorbent used.

#### Deficiency

The deficiency (%) of different gas compositions was calculated based on Eq (2). In this equation, Q_H2S/N2_ (mg H_2_S/g) refers to the adsorption capacity from gas H_2_S/N_2_ and Q_H2S/N2/CO2_ (mg H_2_S/g) refers to the adsorption capacity from gas H_2_S/N_2_/CO_2_.

$$Degradation=\frac{Q_{H2S/N2}-Q_{H2S/N2/CO2}}{Q_{H2S/N2}}x\;100$$ (2)

#### Degradation

The degradation in the adsorption-desorption cycle was calculated as percentage difference using Eq (3) which was based on the previous, and current cycle of the adsorption capacity; where Q_n cycle_ (mg H_2_S/g) refers to the adsorption capacity for the current cycle, whereas Q_n-1 cycle_ (mg H_2_S/g) as the adsorption capacity for the previous cycle.

$$Degradation=\frac{Q_{n\;cycle}-Q_{n-1cycle}}{Q_{n}}x\;100$$ (3)

## Result and discussion

### Characterization analysis for fresh adsorbents

**Fig 2:**
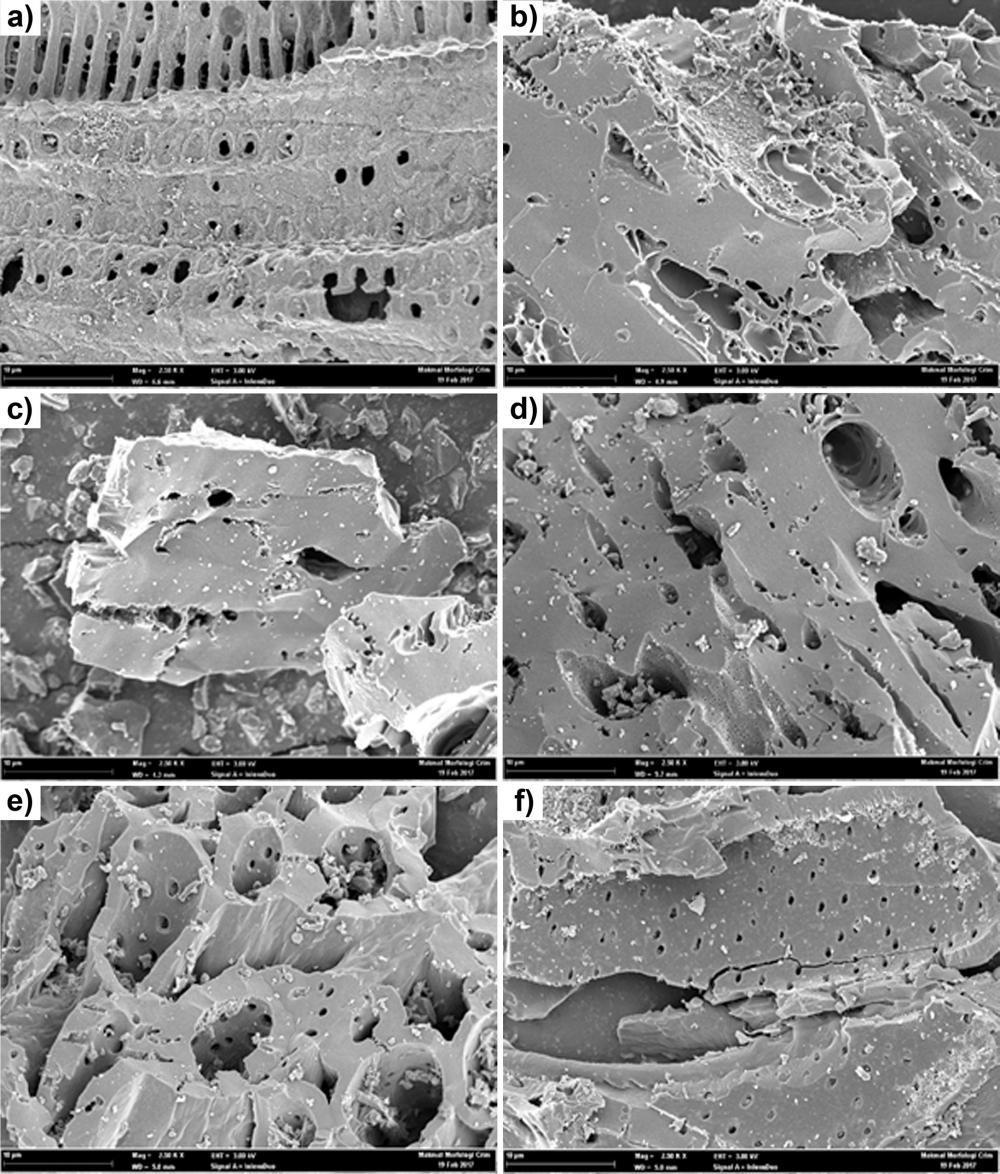
SEM micrograph images of the adsorbent samples at 2.5 k X (10 μm). (a) Raw CAC. (b) KOH–CAC. (c) KI–CAC. (d) CuSO_4_–CAC. (e) Na_2_CO_3_–CAC. (f) ZnAc_2_–CAC.

SEM analysis was performed to observe the morphology and surface structures of synthesized adsorbents. Fig 2 shows the SEM images for fresh, i.e., before the adsorption-desorption process, adsorbent samples. Based on Fig 2, it shows that the external surfaces of the impregnated CACs have cracks, crevices, and some grains in various sizes in a large hole. Then, the EDX detector, attached to the SEM instrument, was employed to estimate the amounts of specific elements on the surface of the adsorbent samples.

SEM images of Na_2_CO_3_–CAC (Fig 2(E)) shows more sponge-like structures, with several small and large holes, than KI–CAC (Fig 2(C)), KOH–CAC (Fig 2(B)), CuSO_4_–CAC (Fig 2(D)), and ZnAC_2_–CAC (Fig 2(F)). The KOH–CAC had a thin film of KOH, which was coated on the CAC surface, where it partially developed as honeycomb-like which highly defined pores and cavities on the surface are. However, the KOH impregnated CACs promoted etching during the activation process that led to the creation of micropores; hence, enhanced the surface area and pore volume of activated carbon.

The ZnAc_2_–CAC sample shows a single fiber, in which each fiber was composed of microfibers. Along with porosity, microfibers make carbon fibers a good material for adsorption and facilitating adsorbents for adsorbate spread. The micrographs also revealed that ZnAc_2_–CAC had a high surface area, and shorter diffusion path, thereby providing a structural foundation for a high specific capacitance [38].

Table 2 shows the EDX elemental analysis for different adsorbents sample. The EDX data shows the elemental analysis of the residue found were C, O, K, I, Na, S, Ca, and Zn in the adsorbents sample. The carbon content of adsorbent decreased after the impregnation with KOH, KI, ZnAc_2_, CuSO_4_, and Na_2_CO_3_, because of the thermal degradation and oxidation of the carbon content. The elements K, I, Zn, S, and Na accumulated on carbon after the impregnation process was found on the surface of CAC.

**Table 2:** Contents of elements (C, O, S, Zn, I, Ca, Na and K) in the fresh adsorbent.

Elements	Raw CAC (wt.%)	ZnAc_2_–CAC (wt.%)	KOH–CAC (wt.%)	KI–CAC (wt.%)	CuSO_4_–CAC (wt.%)	Na_2_CO_3_–CAC (wt.%)
C	80.56	90.18	90.69	84.72	81.34	88.27
O	5.21	3.62	5.52	0.43	7.93	6.95
Cu	0.22	0.08	0	0.52	0.28	0.04
Zn	0.29	4.37	0.39	0.76	0.42	0.02
Na	0.04	0	1.78	0	0	2.91
S	1.13	0.21	0.31	1.02	0.71	0.42
K	6.24	0.47	0.69	3.41	6.81	0.71
Ca	3.19	0.25	0.16	0.44	0.58	0.17

Then, the presence of O elements confirmed that CAC contained functional surface groups, such as carboxyl, carbonyl, and ether groups. In addition, S elements also formed chemicals bonds by the presence of the functional group, such as sulfonate groups in FTIR analysis. Besides, the elements Ca represents in all adsorbents proved the mesoporous materials used are from activated carbon family [39]. However, the contents of impregnated compounds in each of adsorbents was found a small number of elements in terms of wt.%, where, referred to impregnation compounds.

Table 3 indicates the BET surface area (S_BET_) in m^2^/g of the adsorbents sample decreased compared with raw CAC, with the exception of Na_2_CO_3_–CAC. The S_BET_ of the adsorbent increased in the following order: CuSO_4_–CAC < ZnAc_2_–CAC < KI–CAC < KOH–CAC < Raw CAC < Na_2_CO_3_–CAC. The decrease in S_BET_ in the impregnation activated carbon was caused by the blocking of some micropores of the impregnated compound. However, the S_BET_ of Na_2_CO_3_–CAC (901.58 m^2^/g) was slightly higher than raw CAC (901.04 m^2^/g), which was caused by the increase in adsorbents sample porosity during the impregnation of the chemical agent-type alkali carbonate (K_2_CO_3_) [31].

**Table 3:** Porous properties for fresh adsorbents sample from BET analysis.

Adsorbent types	BET surface area, m^2^/g	Total pore volume, m^3^/g (x10^-7^)	V_micro_/V_total_ (%)	Pore size, Ǻ
**Raw CAC**	901.04	4.31	0.74	19.11
**KOH—CAC**	805.45	3.67	0.77	18.23
**KI–CAC**	726.69	3.49	0.76	19.23
**ZnAc**_**2**_**—CAC**	656.75	2.94	0.78	17.93
**Na**_**2**_**CO**_**3**_**—CAC**	901.58	4.28	0.71	18.99
**CuSO**_**4**_**—CAC**	39.76	4.34	0.76	436.14

According to the IUPAC classification of pore dimensions, the pores of adsorbents are divided into three types: micropores (d<2 nm), mesopores (d = 2 nm to 50 nm), and macropores (d>50 nm). Hence, all adsorbents were categorized as micropores, because the pore sizes were below 2 nm, with the exception of CuSO_4_-CAC that was categorized as mesopores. The micropore adsorbents were suitable for gas adsorption [31], whereas larger pore sizes (macro and meso) contribute to molecule capture [40].

Our study obtained a similar trend with those of Sitthikhankaew et al. [29] and Okman et al. [41], where the S_BET_ of the non-impregnated CACs (1343 m^2^/g) was greater than that of impregnated CACs with KOH (1037 m^2^/g). Mt Yusuf et al. [31] also reported the similar pattern as reducing the number of BET surface area of ionic liquid AC (697 m^2^/g– 840 m^2^/g) from raw AC (863 m^2^/g). Moreover, a study on K_2_CO_3_ also reported that the surface area was improved due to the enlargement of the activated carbon porosity [30,42].

Similar to our study, Yusri et al. [43] obtained a low value of BET surface area for Cu materials with S_BET_ around 3–20 m^2^/g, based on wt.% used on mesoporous silicon materials, which was due to mesopore development that occurred with increased metal load; where metal elements (Cu) dispersed, which blocked the interior of pores [44]. Hence, the Cu components have the lowest BET surface area among the impregnated materials.

**Fig 3:**
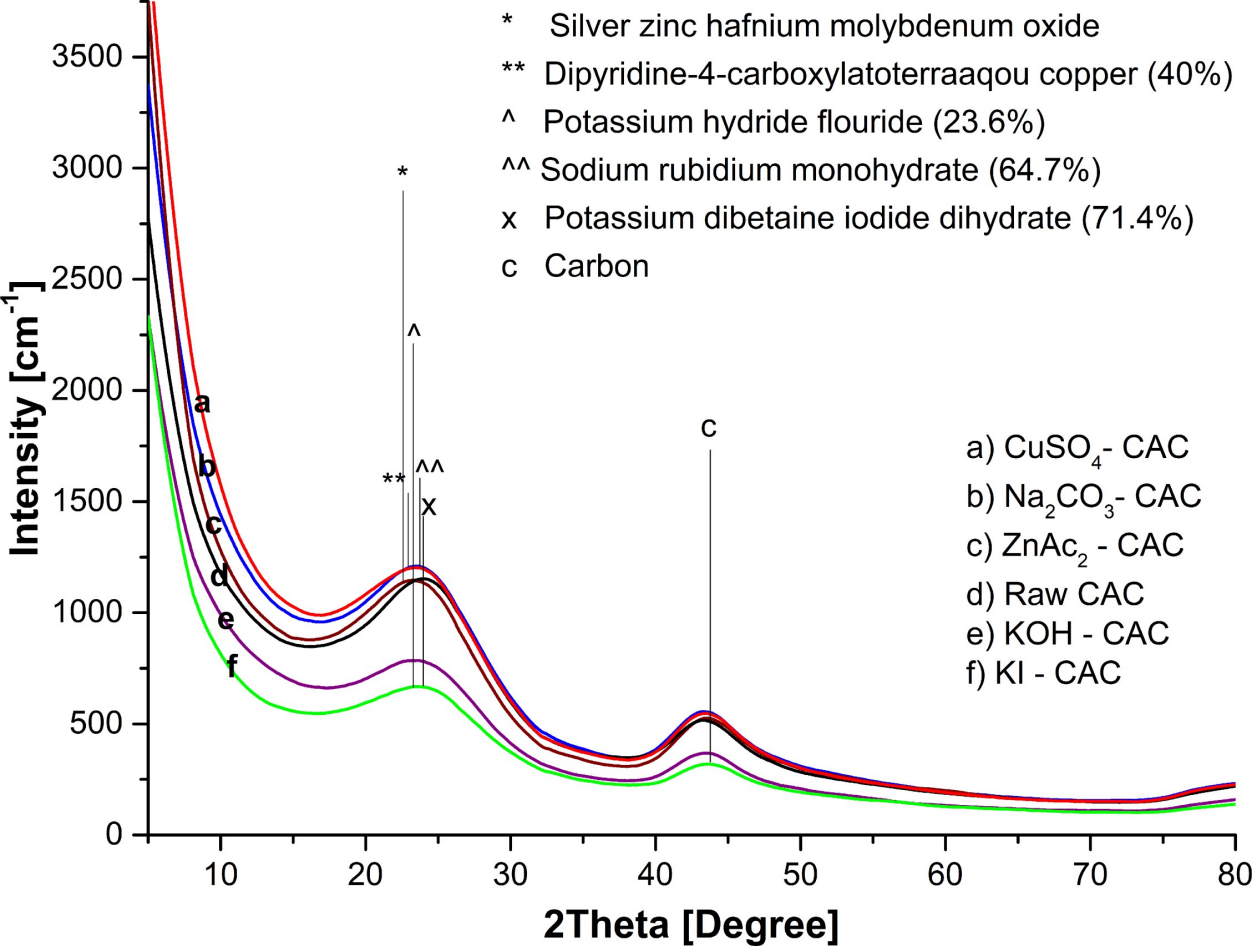
X-ray diffraction (XRD) patterns.

Meanwhile, Fig 3 and Table 4 shows the crystallinity and amorphousness (%) of adsorbent types. Adsorbents were more amorphous than crystalline, which was due to the existence of probable surface functions of activated carbons obtained from agricultural wastes, causing difficulty in crystallization.

**Table 4:** Percentage crystallinity and amorphousness of adsorbents.

Adsorbent types	Crystallinity (%)	Amorphous (%)
**Raw–CAC**	37.8	62.2
**KOH–CAC**	31.3	68.7
**KI–CAC**	26.4	73.6
**ZnAC**_**2**_ **–CAC**	31.7	68.3
**Na**_**2**_**CO**_**3**_**- CAC**	37.0	63.0
**CuSO**_**4**_ **–CAC**	31.6	68.4

The amorphous nature of the activated carbon was determined using the intensity of the observed rays, with respect to the scattering angle (2θ). Fig 3 shows all adsorbent samples as XRD pattern of typical amorphous carbon with broad asymmetric peaks. From this figure, the 2θ positions of the broad peaks, belonging to the composites, have significantly shifted to the left, which indicates the textural, and conformational change associated with the rearrangement of impregnated component chains [45]. The sample of raw CAC shows 2θ = 24.29°, with the intensity of 1151 a.u. of the component carbon at major peaks. Meanwhile, the minor peaks show the component carbon of diamond at 2θ = 44.09°.

The samples of KOH–CAC, KI–CAC, ZnAc_2_–CAC, Na_2_CO_3_–CAC, and Cu_2_SO_4_–CAC, which shifted to left, shows 2θ at 23.86° (potassium hydroxide hydrate), 23.78^o^ (iodine), 24.00° (zinc acetate), 22.84° (sodium carbon oxide), and 24.07° (Djurleite). The peak at 2θ = 23.50° corresponded to the micrographitic structure characteristic of activated carbon. In cases of CAC–KI, the second peaks resulted on potassium iodine at 2θ = 44.09°; similar to the result of Sitthikhankaew et al. [40], which found similar trends for KI at 2θ = 44.50°. Thus, the presence of these minerals explained the adsorbent properties of the adsorbent samples.

High intensity indicated the impregnated component content on activated carbon. The CuSO_4_–CAC shows higher intensity (1422 a.u.) than the raw CAC (1151 a.u.), Na_2_CO_3_–CAC (1252 a.u.), and ZnAc_2_–CAC (1168 a.u.). Hence, the high intensity of CuSO_4_–CAC proved that the CuSO_4_ covered the activated carbon surface the most among another adsorbents sample. The intensity of KOH–CAC (814 a.u.) and KI–CAC (680 a.u.) was lower than that of CAC by about 29.3%.

Similar patterns were observed in the amorphous characteristics of activated carbon at 2θ = 15.00° to 34.00° for raw carbon from raw activated carbon [46] of *Caesalpinia pulcherrima* pods [45], and *Albizia Lebbeck*, *Ziziphus Spina-Christi* seeds [47]. Tangjuank et al. reported that identical XRD spectra with activated carbon were obtained from the cashew nut shells by physicochemical activation (KOH/CO_2_) [47], which proved that activated carbon from agricultural waste is amorphous types because of the existence of probable surface function from the crystallization [48].

**Fig 4:**
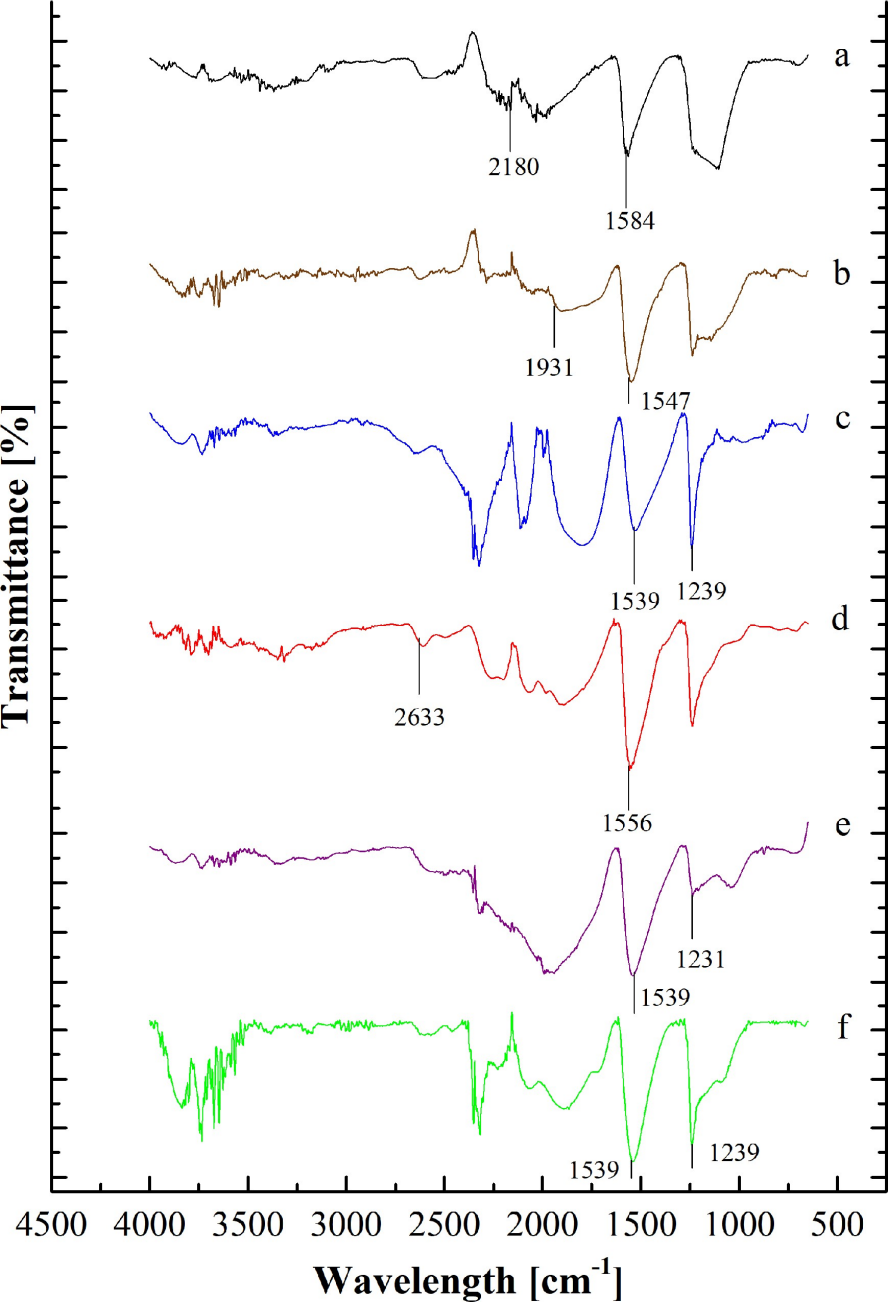
FTIR spectra of adsorbents. (a) Raw CAC (b) ZnAc_2_–CAC (c) Na_2_CO_3_–CAC (d) CuSO_4_–CAC (e) KOH–CAC (f) KI-CAC.

Fig 4 and Table 5 indicate that the FTIR spectra for different adsorbent types. The spectra at ≈900 cm^-1^ and ≈1231 cm^-1^ indicated the existence of alkene (C-H bonds) and carbonyl group (C = O) were for all the adsorbents [48]. Different adsorbents had different functional groups depending on the impregnated material on the activated carbon. KI-CAC obtained the alkyne group (C≡C) at peak ≈2180 cm^-1^ [48].

**Table 5:** Surface functional groups.

Adsorbents	Spectrum wave number (cm^-1^)	Functional group	Reference
**Raw CAC**	≈1230	Carbonyl group (C = O)	[48]
	≈900	Alkene group (C-H bonds)	
**ZnAc**_**2**_**- CAC**	1271–1224	Ether group (R-O-R)	[47]
	3800–3200	Hydroxyl group (O–H)	
**KI-CAC**	≈2180	alkyne group (C≡C)	[48]
	≈1230	Carbonyl Group (C = O)	
**KOH-CAC**	3800–3200	Hydroxyl group (O–H)	[49]
	1550–1200	the carboxylic acid (O–H bonds)	
**CuSO**_**4**_**-CAC**	3800–3200	Hydroxyl group (O–H)	[49]
	≈2633	Sulfonate group (S-H)	
**Na**_**2**_**CO**_**3**_**- CAC**	1271–1224	Ether group (R-O-R)	[53]
	650–600	acetyleric group (C–H bond)	

The presence of oxygen in the impregnated materials, such as KOH, CuSO_4_, Na_2_CO_3_ and ZnAc_2_ obtains difference peaks that indicated carboxylic acid (O–H bonds), ether (stretching C-O-C), hydroxyl (O–H), acid anhydride (RC (= O)OC (= O)R), and aromatic (formation C = C ring) groups at peaks of 1550 cm^-1^ to 1200 cm^-1^, 1271 cm^-1^ to 1224 cm^-1^, 3800 cm^-1^ to 3200 cm^-1^, 1931 cm^-1^ to 1618 cm^-1^ and 1550 cm^-1^ to 1200 cm^-1^ [48]. Besides, the sulfonate (S–O stretch) and acetyleric (C–H bond) group were determined through CuSO_4_, and Na_2_CO_3_ at peaks ≈2633 cm^-1^ and 600 cm^-1^ to 650 cm^-1^ [49].

**Fig 5:**
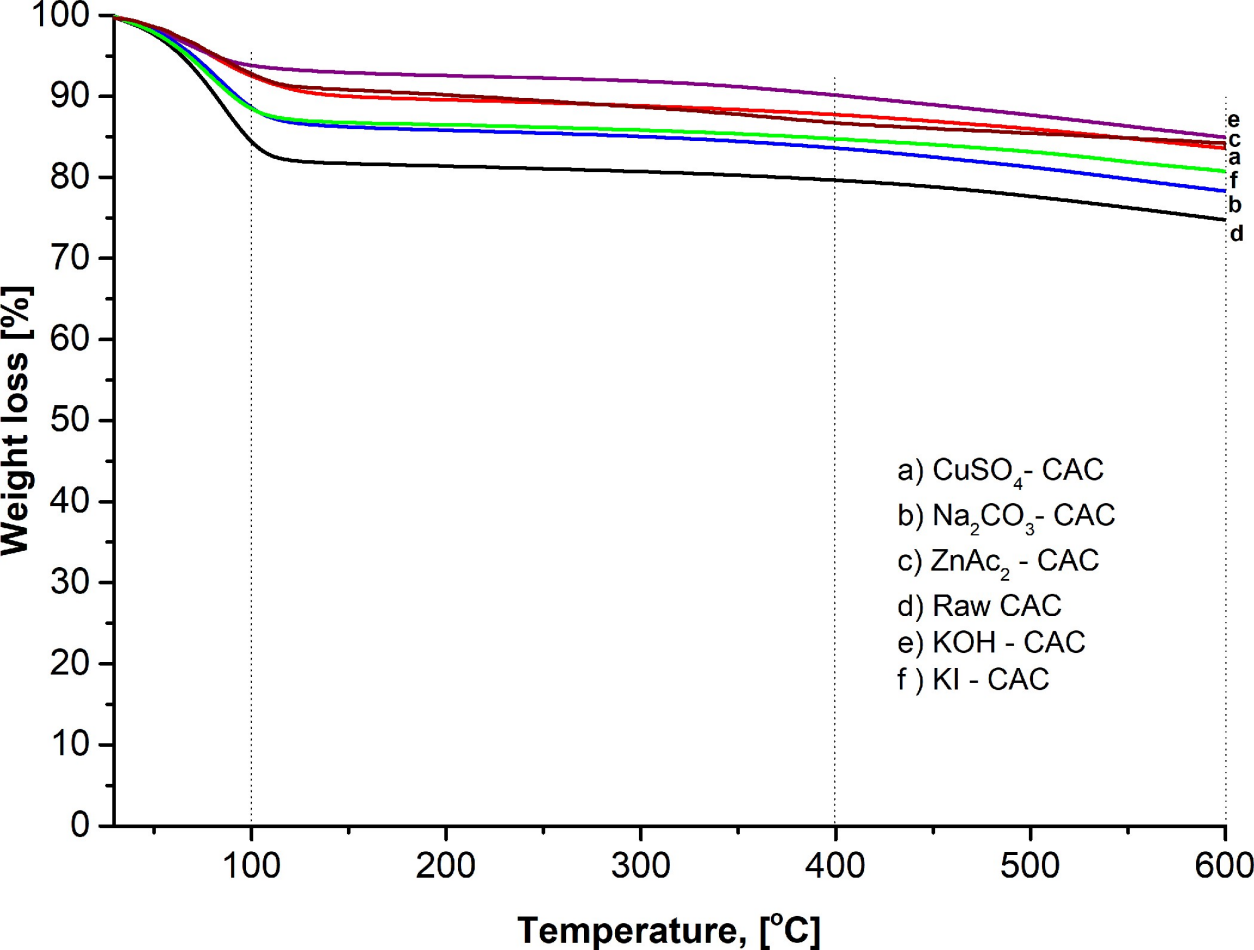
TGA curve for different adsorbent types.

Fig 5 signify the thermal stability of raw CAC and synthesized impregnated CACs. The mass loss during the heating ramp rate was analysed as shown in Table 6. The descending TGA thermal curve of raw and impregnated CACs indicated weight loss (Fig 5). Three stages of thermal decomposition behaviour were determined, despite the three derivative peak temperatures in the TGA curve. The first derivative peak temperature for ZnAc_2_–CAC was 100°C; which indicated that about 7.2% of weight loss occurred from 29°C to 100°C, as shows as in Table 6. The weight loss was attributed to the dried-off moisture in the material. The second derivative peak temperature ranged from 100°C to 400°C, which results in the weight loss of about 12.3%, and decline slightly to the third derivatives (400°C to 600°C) at about 16.4%.

**Table 6:** Adsorbent weight loss at different temperature ranges.

Adsorbents	Temperature range	Weight loss, %
**Raw–CAC**	29°C to 100°C	15.8
	100°C to 400°C	20.4
	400°C to 600°C	25.2
**ZnAc**_**2**_**–CAC**	29°C to 100°C	7.2
	100°C to 400°C	12.3
	400°C to 600°C	16.4
**KOH–CAC**	29°C to 100°C	4.6
	100°C to 400°C	7.0
	400°C to 600°C	10.0
**KI–CAC**	29°C to 100°C	12.4
	100°C to 400°C	15.3
	400°C to 600°C	16.4
**CuSO**_**4**_**–CAC**	29°C to 100°C	9.4
	100°C to 400°C	12.0
	400°C to 600°C	14.1
**Na**_**2**_**SO**_**3**_**–CAC**	29°C to 100°C	12.8
	100°C to 400°C	16.0
	400°C to 600°C	18.7

In general, a small weight loss was observed at a temperature higher than 200°C. However, the decomposition and association continue. The largest weight loss of the materials occurred during carbonization; by contrast, no significant weight loss occurred during activation. All adsorbent samples had about 4% to 16% of weight loss at the earlier stage (29°C to 100°C). Other researchers also determined that raw and impregnated CACs had weight losses of about 20% and 50%, respectively, at 500°C in the elimination of the moisture by the volatile compound [50].

Dehydration also increased sharply with impregnation and resulted in the minor release of the volatile materials. Therefore, the increment of available impregnated components improved adsorption performance, which leads to a good porous solid.

### Adsorbent performance

The adsorbent performance was tested by using commercial mixed gas subjected to different operating parameters, such as flow rate, length bed used, type of gases used and the adsorbents. In order to determine the adsorption capacity, the breakthrough and saturated points indicate when the H_2_S concentrations reach 1 ppm and 1000 ppm at the outlet stream, respectively. Moreover, the data were represented as an average result from three (3) times of repetitions.

#### Effect of feed flow rate with H_2_S/N_2_ gas

**Fig 6:**
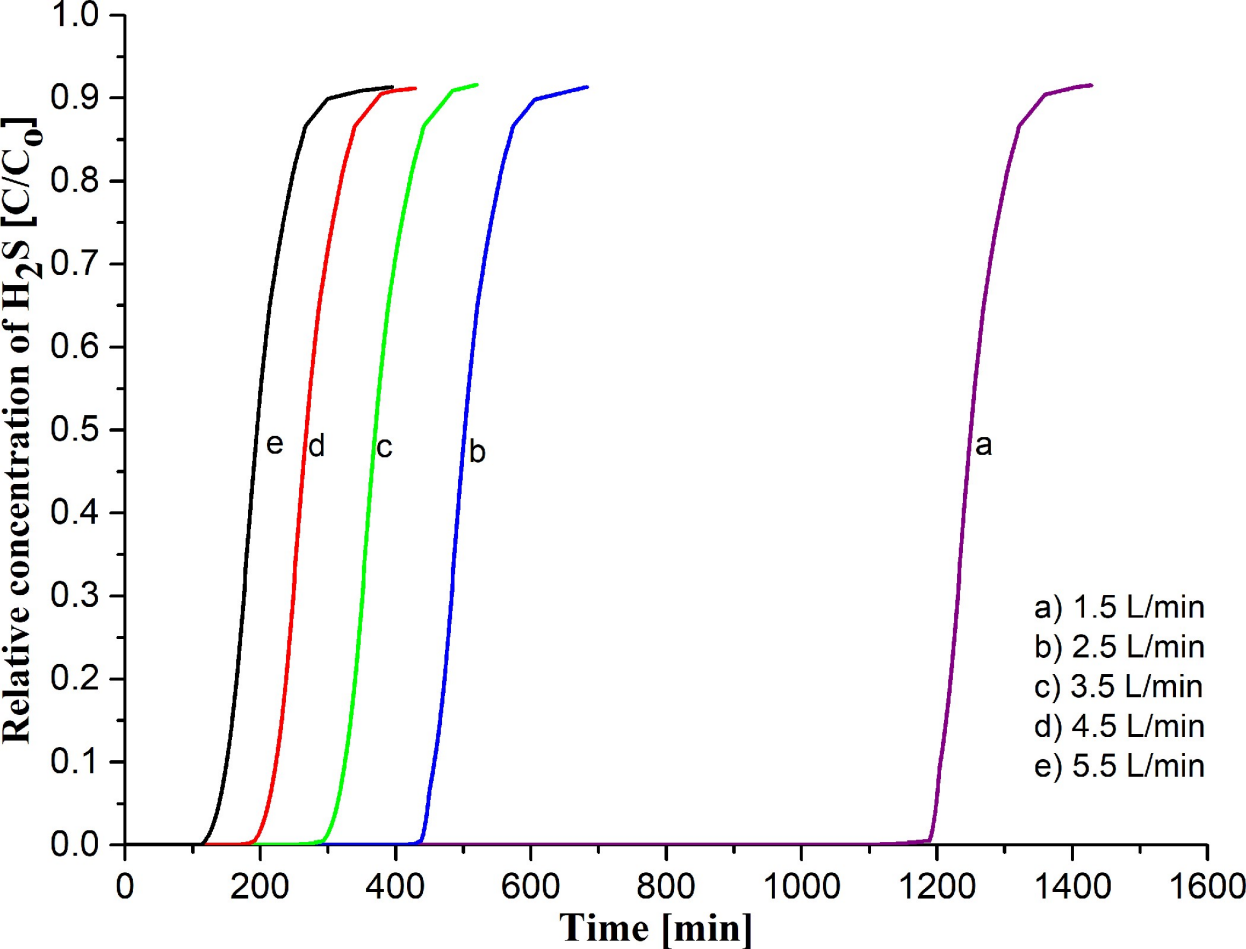
The breakthrough curve on the effect of feed flow rate using raw CAC with H_2_S/N_2_ feed.

Different flow rates affected adsorbent capacity for H_2_S gas. Fig 6 illustrates the breakthrough profile for the different feed flow rate of H_2_S/N_2_ using Raw CAC as adsorbent at L/D = 2.5, where 0.155 kg of adsorbents loaded into the adsorber column. As shown in Fig 6, the profile curve increased exponentially with S curve, before being nearly constant at 1.0, due to the saturated H_2_S. The profile curve was similar for all feed flow rate. Meanwhile, the adsorption capacity calculated based on Eq (1). Overall, the performance of different feed flow rate had affected the adsorption capacity because of breakthrough time periods.

The feed flow rate at 5.5 L/min had more adsorption capacity (1.637 mg H_2_S/g) than 1.5 L/min (0.584 mg H_2_S/g), which was attributed to the longer time required for a breakthrough. As a result, a higher gas flow rate affects the mass transfer zone to achieve the exit column by blocking the active site with the H_2_S gas rapidly [51]. The effects of flow rate to the adsorption of H_2_S on activated carbon results were similar to the study of Natalie [52]. Because of the economic points of gas usage and practicality of use on an industrial scale, the highest flow rate was used for further studies. The breakthrough time and adsorption capacity decreased due to the raised feed flow rate [53].

#### Effects of the L/D ratio with H_2_S/N_2_

**Fig 7:**
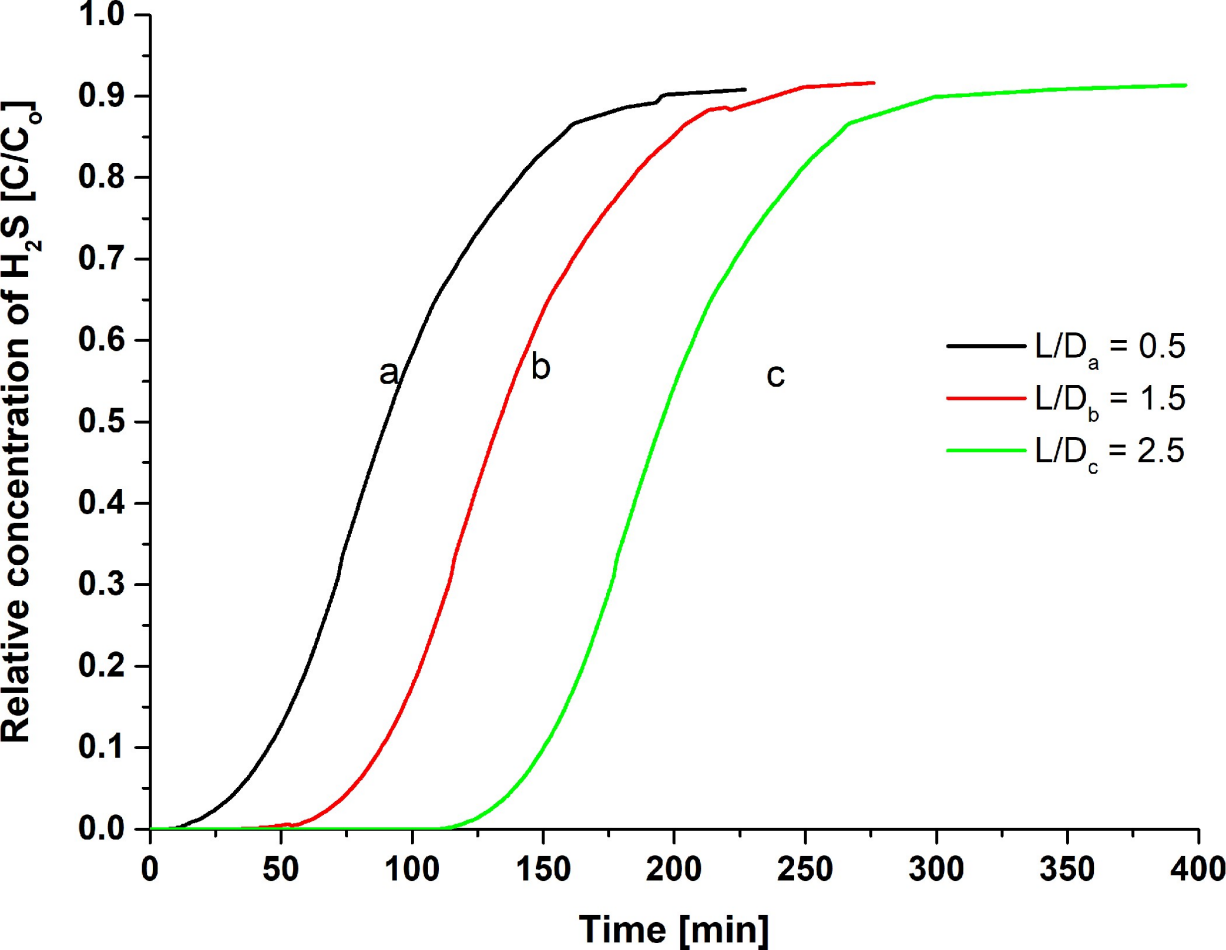
The breakthrough curve on the effect of L/D ratio for raw CAC as an adsorbent with H_2_S/N_2_ feed.

Three beds with different L/D ratio were used to determine the adsorption capacity effect by using raw CAC at a constant feed flow rate (5.5 L/min). The L/D ratio used were 0.5, 1.5, and 2.5 instead of 5.0, due to the longer breakthrough time even at a bigger feed flow rate which commonly uneconomical for the lab scale operation. Different L/D ratio resulted in different mass adsorbent loaded (0.052 kg, 0.103 kg and 0.155 kg) in different length bed used. Hence, the result shows as in Fig 7 and Table 7 respectively. The breakthrough time and L/D ratio were positively correlated with amounts of adsorbents loaded into the column. Low amounts of carbon loaded indicated a low number of active sites on raw CAC surface volume which indicate low possibility in capturing H_2_S gas. The L/D = 2.5 resulted in a longer breakthrough time (109 min) than the L/D = 0.5 (8 min). Natalie [52] also reported the higher amounts of adsorbents loaded resulted for a longer breakthrough time which indicate the effective H_2_S removal.

**Table 7:** H_2_S adsorption performance at difference lengths of bed used.

L/D ratio	Breakthrough time, min	Saturation time, min	Adsorption capacity at 1 ppm, mg H_2_S/g
0.5	8	13.5	0.128
1.5	35	51.5	0.284
2.5	109	127.5	0.584

#### Effect of adsorbent types and gas composition

**Fig 8:**
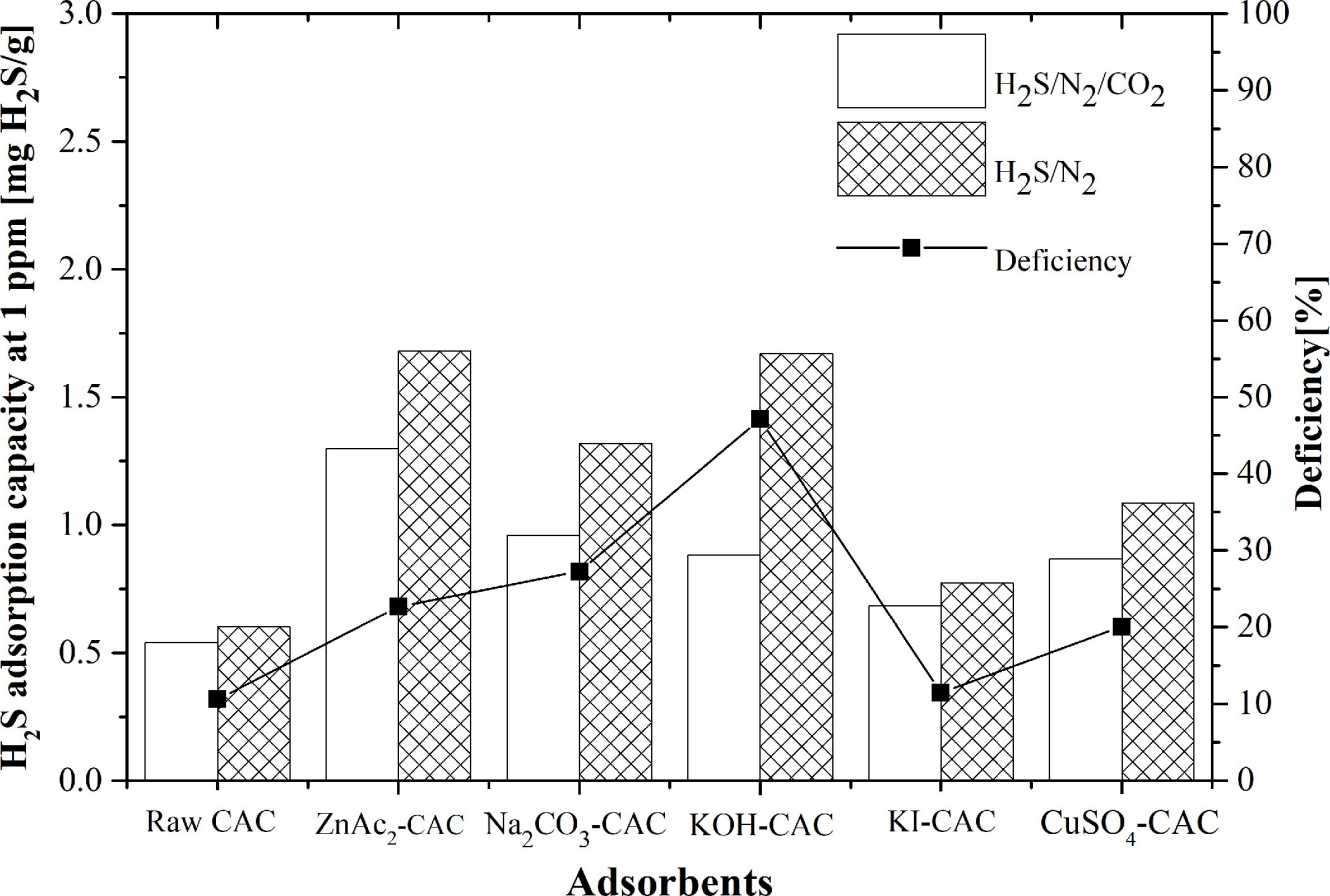
Comparison of adsorption capacity between H_2_S/N_2_ and H_2_S/N_2_/CO_2_ gas composition with their deficiency.

The H_2_S/N_2_ gas and H_2_S/N_2_/CO_2_ (mixed gas) had different adsorption capacities. The adsorption occurred as physical adsorption, which started with the bulk stream of H_2_S transferred onto the surface of the adsorbents. Fig 8 illustrates the adsorption performance using different types of adsorbent at ambient conditions, gas composition (H_2_S/N_2_ and H_2_S/N_2_/CO_2_ gas) and feed flow rate at 5.5 L/min. The outlet H_2_S concentration increased with S curve at the breakthrough point at 0.001 and was nearly constant at 1.0 due to the saturated H_2_S for both types of gases. The breakthrough time found a significant difference for both gas compositions was due to the presence of CO_2_ gas.

Based on Fig 8, the feed gas composition with H_2_S/N_2_ shows the ZnAc_2_-CAC had higher adsorption capacity compared with other adsorbents. The adsorption capacity of ZnAc_2_-CAC and raw CAC was 1.68 mg H_2_S/g and 0.582 mg H_2_S/g, respectively; which was caused by attraction surface of ZnAc_2_-CAC which provided a structural foundation for high specific capacitance [53]. Sitthikhankaew et al. [29] proved that the adsorption capacity for the impregnated CACs was higher than that of raw CAC. Besides, KOH, KI, and Na_2_CO_3_ caused the adsorption capacity increase from 1.56 mg H_2_S/gram to 1.58 mg H_2_S/gram, with breakthrough time ranging from 109 min to 151 min. KOH–CAC had longer breakthrough time than KI–CAC, and Na_2_CO_3_–CAC; similarly, the study of Cui et al. [54] in the synthetic natural gas produced by petroleum also result in the same pattern.

Although, the fed composition gas of H_2_S/N_2_/CO_2_ had slightly differed from H_2_S/N_2_ composition due to the presence of CO_2_. Significantly, the raw CAC prove to be the lowest capability in capturing the H_2_S and CO_2_ gas among the impregnated CACs. The increase in the H_2_S adsorption capacity of the impregnated CACs was caused by the large surface area and porosity development. The presence of CO_2_ gas in the outlet stream had decreased the H_2_S adsorption capacity due to the competition for both gases (CO_2_ and H_2_S) on the raw CAC active site surface, which led to the limitation of H_2_S adsorption through the formation of high sulfur-valent components [39].

The breakthrough curves were illustrating the adsorption performance as in the order: ZnAc_2_-CAC (1.293 mg H_2_S/g)>Na_2_CO_3_-CAC (0.953 mg H_2_S/g)>KOH–CAC (0.878 mg H_2_S/g)>KI–CAC (0.673 mg H_2_S/g)>CuSO_4_–CAC (0.539 mg H_2_S/g)>raw CAC (0.528 mg H_2_S/g), which different from those of the H_2_S/N_2_ gas composition, in which the second preferable impregnated CACs was Na_2_CO_3_–CAC, instead of KOH–CAC. This phenomenon indicated that the presence of CO_2_ in the mixed gas was blocked on the CAC surface.

The different adsorbents had different characteristics, which indicated that specific adsorbed gas was suitable for certain adsorbents. Hence, the CO_2_ gas more preferable to attach on the CuSO_4_–CAC, KOH–CAC, Na_2_CO_3_–CAC, ZnAc_2_–CAC, KI–CAC, and raw CAC surfaces because of the huge difference in the adsorption capacity. The capability of adsorbents had increased the affinity of CO_2_ molecules to reside on the surface. As reported by Soraya et al. [55], the component Cu adsorbed more CO_2_ gas than component Zn.

Fig 8 also exemplify the adsorption deficiency capacity of H_2_S between two different types gas compositions, based on the calculation in Eq (2), in order: CuSO_4_–CAC (49.7%)>KOH–CAC (46.7%)>Na_2_CO_3_–CAC (27.5%)>ZnAc_2_–CAC (23%)>KI–CAC (21.4%)>raw CAC (9.3%). Samanta et al. [56] concluded the enhancement of the CO_2_ adsorption capacity was caused by the reaction of CO_2_ with the supporting metals that occurred during the physical adsorption onto the micropores of carbon.

Na_2_CO_3_–CAC also reacted with the CO_2_ gas, which led to an increase in the CO_2_ adsorption capacity [57]. However, the Na species and their dispersion, which are directly related to the surface chemistry of the host carbon material, affected the adsorption capacity and stability. Sreńscek-Nazzal et al. [58] found that the impregnated CACs with KOH had maximum CO_2_ adsorption capacity, which led to higher difference than that of the H_2_S adsorption deficiency.

The breakthrough curve proved that the H_2_S gas was preferable on the ZnAc_2_–CAC surface than on other adsorbents. Therefore, the decrease in the H_2_S adsorption capacity was reflected from the CO_2_ adsorption onto the micropores.

### Adsorption-desorption cycle

**Fig 9:**
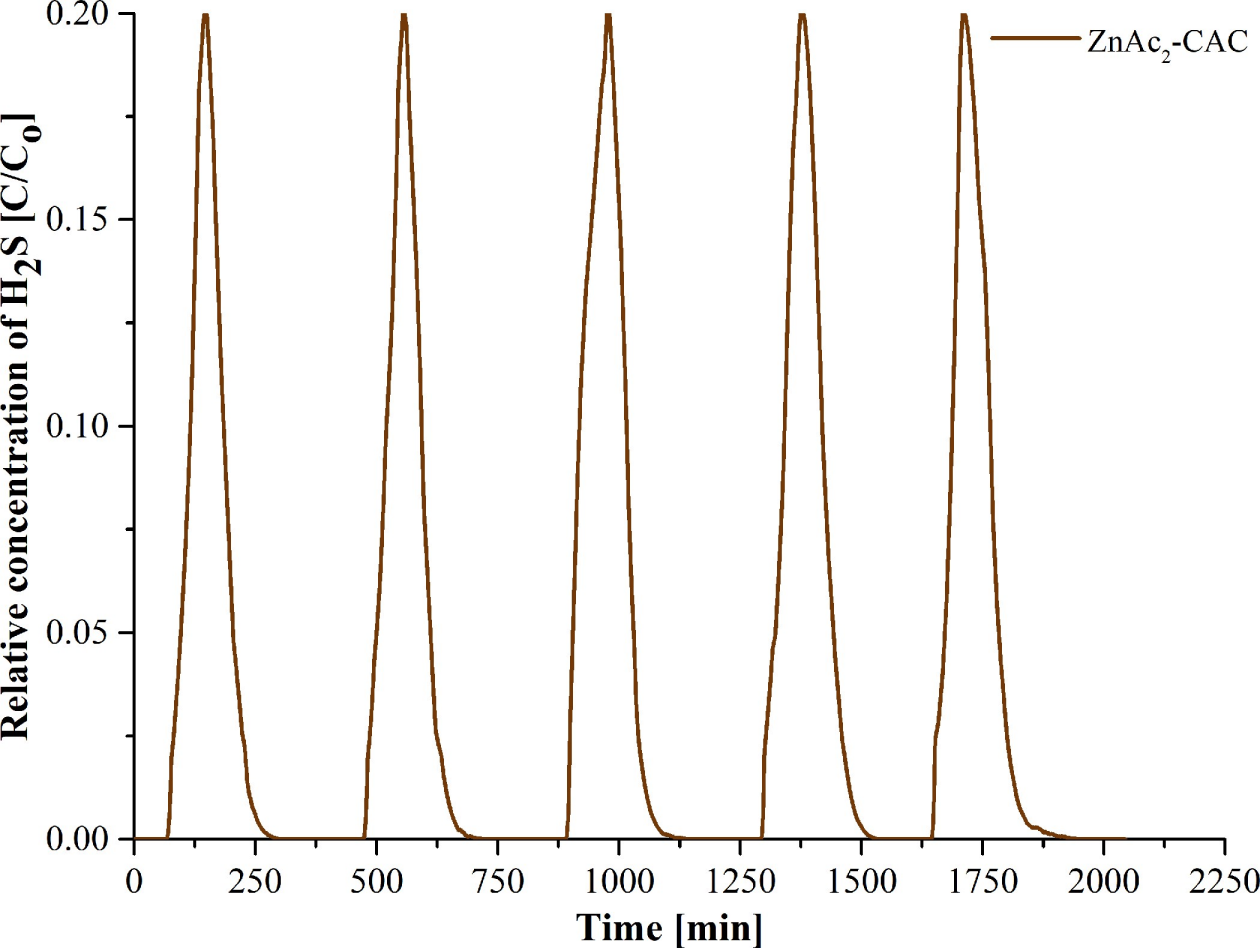
Adsorption-desorption cycle profile for H_2_S removal using ZnAc_2_-CAC.

In this study, the adsorption-desorption cycle established ZnAc_2_-CAC as the best adsorbent in capturing the H_2_S gas for both gasses’ composition (H_2_S/N_2_ and H_2_S/N_2_/CO_2_); using high concentrations (5000 ppm) of H_2_S, instead of 1000 ppm due to the economic and shorten the operating period. Fig 9 illustrates the adsorption-desorption (regeneration) cycle profiles for ZnAc_2_-CAC.

Fig 9 and Table 8 show that ZnAc_2_-CAC maintained the H_2_S adsorption capacity at 1.831 mg H_2_S/g for the first three cycles; then, continuously degraded on the next cycles. From the fourth cycle onwards, the capability of the adsorbents dropped, which was calculated based on Eq (3). The degradation of the adsorbent capability was due to the presence of sulfur elements which blocked the active site on the surface of the adsorbents [59]; thus, limiting the number of regeneration cycles. Other studies suggested using a higher temperature, i.e., 200°C to 250°C, to overcome the degradation of adsorption capacity.

**Table 8:** Regeneration performance ZnAc_2_-CAC.

Number of cycles	Breakthrough time at 1 ppm, min	Adsorption capacity, mg H_2_S/g	Degradation, %
1	68	1.831	0
2	68	1.831	0
3	68	1.831	0
4	65	1.750	4.4
5	63	1.697	7.3

### Characterization of adsorption-desorption adsorbents

**Fig 10:**
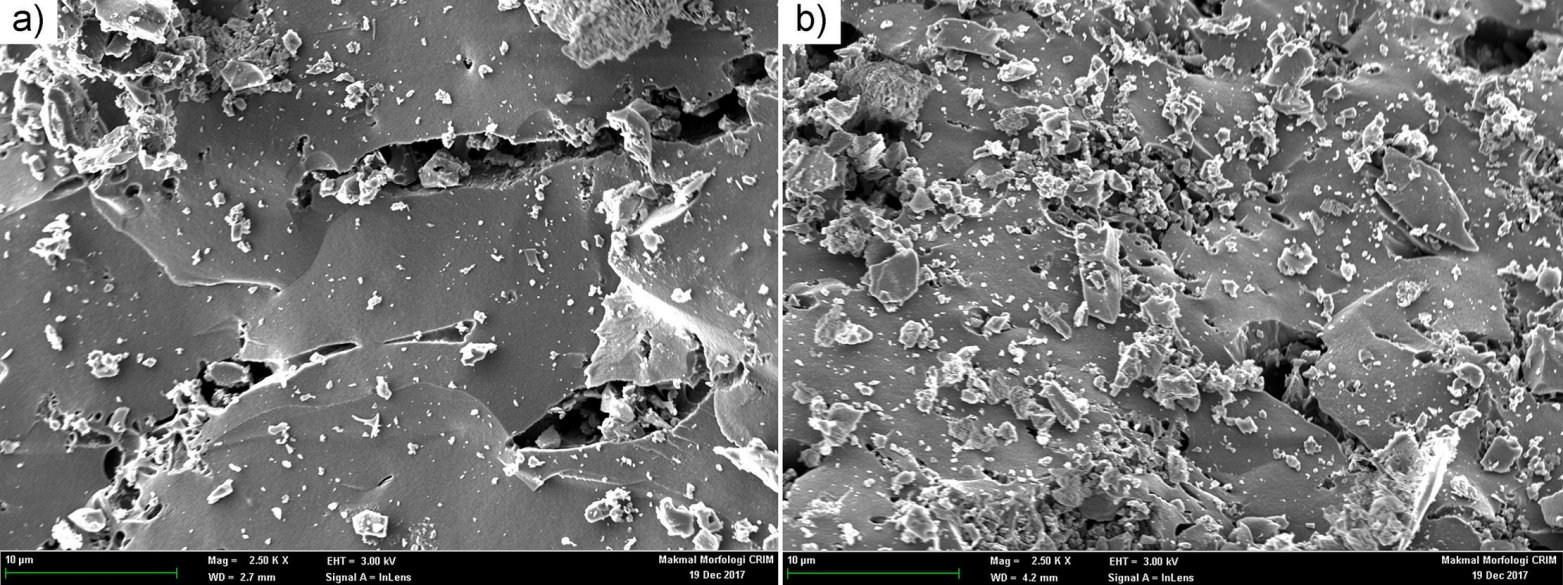
SEM micrograph image of the adsorbents at 2.5 k X (10 μm) (a) ZnAc_2_–CAC_A; (b) ZnAc_2_–CAC_D.

Fig 10 shows the SEM images for saturated (ZnAc_2_-CAC_A) and desorption (ZnAc_2_-CAC_ D). The saturated sample, i.e., adsorbent with H_2_S content more than 1000 ppm, and the desorption sample, i.e., adsorbent after the desorption process, were analysed during the 5^th^ adsorption-desorption cycle. The specific elements were determined through the EDX detector as presents in Table 9.

**Table 9:** Contents of elements in the regeneration of ZnAc_2_–CAC sample.

Elements	ZnAc_2_–CAC_A (wt.%)	ZnAc_2_–CAC_D (wt.%)
C	84.3	95.4
O	6.3	3.9
K	1.1	0.2
S	5.7	0.1
Zn	2.0	0.3

From Table 9, it is proved that the impregnated materials and gas elements were detected on the surface of CAC impregnation. Moreover, the presence of sulfur, S, elements at the EDX data confirmed that H_2_S adsorption occurred on the surface of CAC during the adsorption process. Besides, the elements S can be seen also on the surface of CAC during adsorption and desorption process, respectively. A similar finding was obtained by by Isik-Gulsac [39], which stated that also had the components of H_2_S had occupied on the surface of adsorbents. This condition would affect the degradation of wt.% of carbon as shown in Table 9, due to the H_2_S gas had covered up on the CAC surface area which affected to drop the adsorbent capability. This process can be seen through the adsorption-desorption cycles started at third cycle an upwards. The good performance in H_2_S captured through ZnAc_2_-CAC shows the adsorbents contains higher S elements (5.7 wt.%) compared to desorption adsorbents (0.1 wt.%) as mentioned in Table 9 (ZnAc_2_-CAC_A and ZnAc_2_-CAC_D).

Table 10 shows the BET results of saturated adsorbents (ZnAc_2_-CAC_A) and desorption adsorbents (ZnAc_2_-CAC_D). Generally, the saturated and desorption adsorbents properties were changed in the porous textures and the correlation between nitrogen adsorption-desorption measurements and sulfur adsorption data.

**Table 10:** Porous properties for regeneration of ZnAc_2_–CAC sample from BET analysis.

Adsorbent types	BET surface area, m^2^/g	Total pore volume, m^3^/g (x10^-7^)	V_micro_/V_total_ (%)	Pore size, Ǻ
ZnAc_2_–CAC_A	625.43	3.01	0.79	19.26
ZnAc_2_–CAC_D	717.41	3.48	0.77	19.41

Based on Table 10, the S_BET_ for saturated and desorption adsorbents decreased compared to that fresh adsorbents due to the occupied of impregnated material and H_2_S gases on the CAC surface. However, the low value of S_BET_ resulted in the higher adsorption capacity. Meanwhile, the ZnAc_2_-CAC_D showed a higher value of S_BET_ compared to fresh and saturated (ZnAc_2_-CAC_A) adsorbent which end up affected towards adsorption capability at third cycle and upwards. This phenomenon related to the distribution of H_2_S gas components on the surface of CAC during the cycle process. Moreover, the reduction of total pore volume strongly support the H_2_S gas components attached on the CAC surface. These tendencies were similar to Sitthikhankaew et al. [28, 29] which obtained a similar finding on the decrease in S_BET_ for impregnated CACs compared to the Raw CAC.

**Fig 11:**
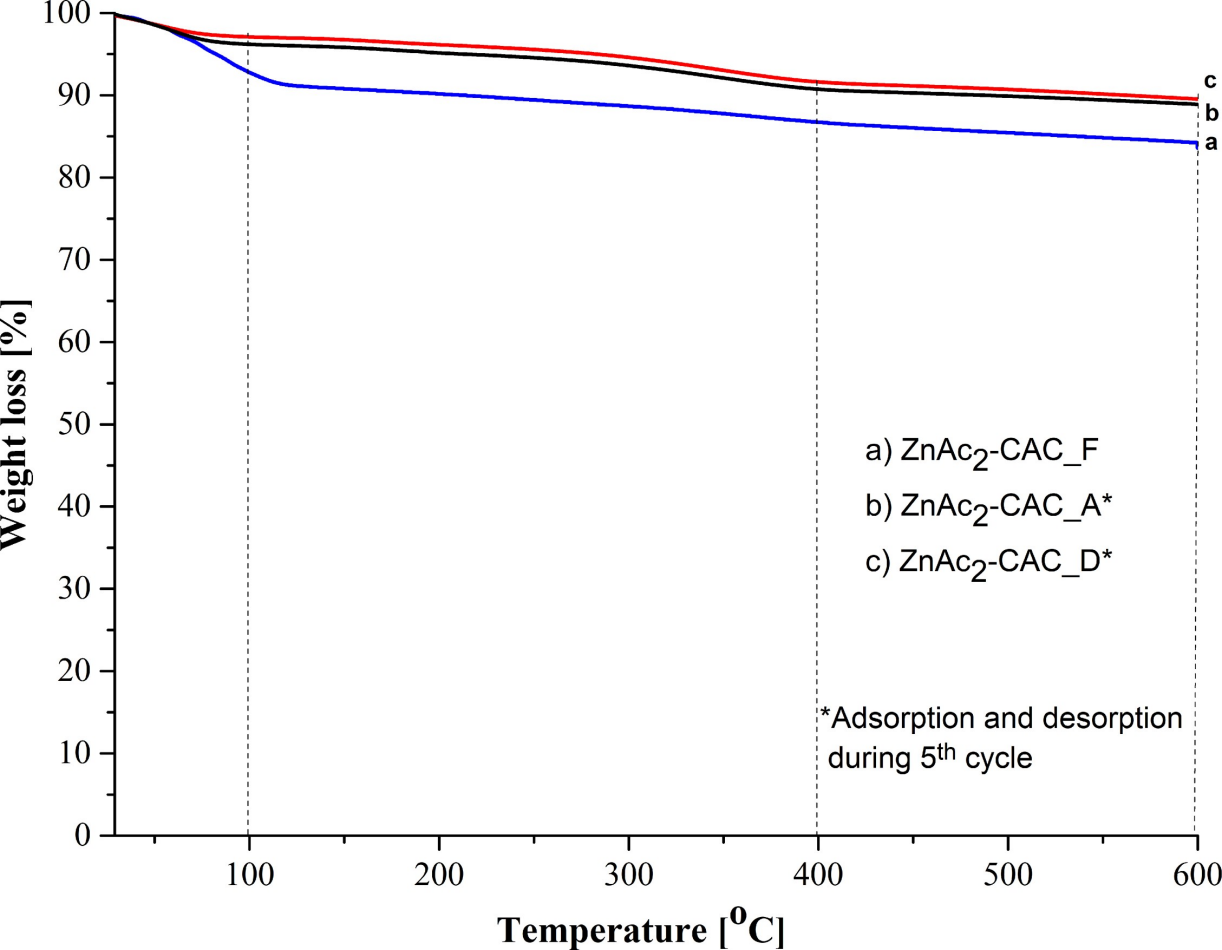
Thermal profile for regeneration of ZnAc_2_–CAC sample.

In the case of the thermal analysis, Fig 11 shows the thermal stabilization curve of the adsorbent for saturated (ZnAc_2_-CAC_A) and desorption (ZnAc_2_-CAC_D) adsorbents. Similar to previous analysis for fresh adsorbents, the analysis based on three thermal derivatives by observing the weight lost from 32°C to 600°C. It was easily understood that, the first temperature derivative 32°C—100°C is known for moisture loss that naturally contains in the adsorbents.

As mentioned previously, the fresh ZnAc_2_-CAC cannot withstand with high temperature during the adsorption process. However, in the desorption process for the regeneration study, the performance of the adsorbent at first and second derivative remained similar with fresh adsorbents even after adsorption and desorption process. Anyhow, the present of adsorbate on adsorbents surface during the adsorption-desorption process may influence on adsorption capability for next cycle of regeneration [60].

Although in the adsorption process by fresh adsorbents requires a low temperature, however, it would be different for the regeneration process, which needs probably high temperature in order to eliminate the adsorbates on the surface CAC. These statements agreed with Feng et al. [61], where high temperature for elimination and broken down the strong S-bond on the surface adsorbents are needed. Hence, the ZnAc_2_-CAC had own specific optimum temperature to use in adsorption (32°C to 400°C) while, desorption needs high temperature up to 600°C.

## Conclusion

The impregnated CACs resulted in good capability in capturing H_2_S gas through several operating parameters such as flow rate, adsorbents type, L/D adsorber ratio and different gas composition compared to raw CAC. The impregnated CACs found 58–64% (ZnAc_2_-CAC) much better to adsorp H_2_S gas compared to raw CAC. Besides, characterizations of the fresh adsorbents were gathered as evidence to support the chemical and physical characteristic of the adsorbent and a guidance in preparing the adsorption-desorption operational. It showed that the ZnAc_2_-CAC has capable to use as H_2_S adsorbent for the adsorption-desorption process in several cycles. Besides, the characterizations for post-adsorption-desorption adsorbents had been done to support the behaviour of adsorbents capability degradation in the continuous system. Thus, it is suggested to improve the desorption technique to prevent further degradation on the H_2_S adsorbent. Hence, it will maintain the capability of H_2_S adsorption throughout several cycles of the adsorption-desorption process for biogas purification.

## Supporting information

S1 Fig SEM micrograph image of the adsorbents at 2.5 k X (10 μm) (a) ZnAc2–CAC_A; (b) ZnAc2–CAC_D.
(PDF)

S1 Table Contents of elements (C, O, S, Zn, I, Ca, Na, and K) in the fresh adsorbent. (PDF)

S2 Table Porous properties for fresh adsorbents sample from BET analysis. (PDF)

S3 Table Percentage crystallinity and amorphousness of adsorbents. (PDF)

S4 Table Porous properties for regeneration of ZnAc_2_–CAC sample from BET analysis. (PDF)

S1 File X-ray diffraction (XRD) patterns. (OPJ)

S2 File FTIR spectra of adsorbents. (OPJ)

S3 File TGA curve for different adsorbent types. (OPJ)

S4 File The breakthrough curve on the effect of feed flow rate using raw CAC with H_2_S/N_2_ feed. (OPJ)

S5 File The breakthrough curve on the effect of L/D ratio for raw CAC as an adsorbent with H_2_S/N_2_ feed. (OPJ)

S6 File Comparison of adsorption capacity between H_2_S/N_2_ and H_2_S/N_2_/CO_2_ gas composition with their deficiency. (OPJ)

S7 File Adsorption-desorption cycle profile for H_2_S removal using ZnAc_2_-CAC. (OPJ)

S8 File Thermal profile for regeneration of ZnAc_2_–CAC sample. (OPJ)

## References

[1] Gulagi A, Choudhary P, Bogdanov D, Breyer C. Electricity system based on 100% renewable energy for India and SAARC. *PLoS ONE*. 2017; 12(7): e0180611 10.1371/journal.pone.0180611. 28723937 PMC5516989

[2] Zulkefli N.N, Masdar M.S, Jahim J, Harianto E. H. Overview of H_2_S removal technologies from biogas production. *Int*. *J*. *of Applied Engineering Research*. 2016; 11:10060–10066.

[3] Rasi R, Veijanen A. & Rintala J. Trace compounds of biogas from different biogas production plants. *Energy*. 2007; 32(8): 1375–1380. 10.1016/j.energy.2006.10.018.

[4] Rasi S, Läntelä J. & Rintala J. Trace compounds affecting biogas energy utilisation–A review. *Energy Conversion and Management*. 2011; 52 (12): 3369–3375. 10.1016/j.enconman.2011.07.005.

[5] Papurello D, Boschetti A, Silvestri S, Khomenko L. & Biasioli F. Real-time monitoring of removal of trace compounds with PTR-MS: Biochar experimental investigation. *Renewable Energy*. 2018; 125: 344–355. 10.1016/j.renene.2018.02.122.

[6] Papurello D, Silvestri S, Tomasi L, Belcari I, Biasioli F. & Santarelli M. Biowaste for SOFCs. *Energy Procedia*. 2016; 101: 424–431. 10.1016/j.egypro.2016.11.054.

[7] Alma T, José M. E, Lebrero R. & Muñoz R. A comparative analysis of biogas upgrading technologies: Photosynthetic vs physical/chemical processes. *Algal Research*. 2017; 25: 237–243.

[8] Chambers A. K. and Potter I. Gas utilization from sewage waste, carbon and energy management. *Alberta Research Council*. 2002.

[9] Liu M, Kleij A. V. D, Verkooijen A.H.M. & Aravind P.V. An experimental study of the interaction between tar and SOFCs with Ni/GDC anodes. *Applied Energy*. 2013;108: 149–157. 10.1016/j.apenergy.2013.03.020.

[10] Cavallia A, Kunzeb M. & Aravind P. V. Cross-influence of toluene as tar model compound and HCl on Solid Oxide Fuel Cell anodes in Integrated Biomass Gasifier SOFC Systems. *Applied Energy*. 2018; 231: 1–11. 10.1016/j.apenergy.2018.09.060.

[11] Papurello D, Lafrate C, Lanzini A. & Santarelli M. Trace compounds impact on SOFC performance: Experimental and modelling approach. *Applied Energy*. 2017; 208,:637–654. 10.1016/j.apenergy.2017.09.090.

[12] Kupecki J, Papurello D, Lanzini A, Naumovich Y, Motylinski K, Blesznowski M. & Santarelli M. Numerical model of planar anode supported solid oxide fuel cell fed with fuel containing H_2_S operated in direct internal reforming mode (DIR-SOFC). *Applied Energy*. 2018; 230: 1573–1584. 10.1016/j.apenergy.2018.09.092.

[13] Papurello D. & Lanzini A. SOFC single cells fed by biogas: Experimental tests with trace contaminants. *Waste Management*. 2018; 72: 306–312. 10.1016/j.wasman.2017.11.030. 29158002

[14] Madi H, Lanzini A, Papurello D, Diethelm S, Ludwig C, Santarelli M. & Herle J.V. Solid oxide fuel cell anode degradation by the effect of hydrogen chloride in stack and single cell environments. *Journal of Power Sources*. 2016; 326: 349–356. 10.1016/j.jpowsour.2016.07.003.

[15] Madi H, Lanzini A, Diethelm S, Papurello D, Herle J. V, Matteo Lualdi M, Larsen J. G. & Santarelli M. Solid oxide fuel cell anode degradation by the effect of siloxanes. *Journal of Power Sources*. 2015; 279: 460–471. 10.1016/j.jpowsour.2015.01.053.

[16] Mulbry W, Selmer K, Lansing S. Effect of liquid surface area on hydrogen sulfide oxidation during micro-aeration in dairy manure digesters. *PLoS ONE*. 2017; 12(10): e0185738 10.1371/journal.pone.0185738 28976998 PMC5627928

[17] Miltner M, Makaruk A. & Harasek M. Review on Available Biogas Upgrading Technologies and Innovations Towards Advanced Solutions. *Journal of Cleaner Production*. 2017.

[18] Ryckebosch E, Drouillon M, Vervaeren H. Techniques for transformation of biogas to biomethane. *Biomass and Bioenergy*. 2011; 35(5): 1633–1645. 10.1016/j.biombioe.2011.02.033

[19] Papurello D, Silvestri S. & Lanzini A. Biogas cleaning: Trace compounds removal with model validation. *Separation and Purification Technology*. 2019; 210: 80–92. 10.1016/j.seppur.2018.07.081.

[20] Lanzini A, Madi H, Chiodo V, Papurello D, Maisano S, Santarelli M. & Herle J. V. Dealing with fuel contaminants in biogas-fed solid oxide fuel cell (SOFC) and molten carbonate fuel cell (MCFC) plants: Degradation of catalytic and electro-catalytic active surfaces and related gas purification methods. *Progress in Energy and Combustion Science*. 2017; 61: 150–188. 10.1016/j.pecs.2017.04.002.

[21] Papadias D. D. & Ahmed S. & Kumar R. Fuel quality issues with biogas energy–An economic analysis for a stationary fuel cell system. *Energy*. 2012; 44(1): 257–277. 10.1016/j.energy.2012.06.031.

[22] Barelli L, Bidini G, Arespacochaga N. D, Pérez L. & Sisani E. Biogas use in high temperature fuel cells: Enhancement of KOH-KI activated carbon performance toward H_2_S removal. *International Journal of Hydrogen Energy*. 2017; 42(15): 10341–10353. 10.1016/j.ijhydene.2017.02.021.

[23] Barelli L, Bidini G, Desideri U, Discepoli G. & Sisani E. Dimethyl sulfide adsorption from natural gas for solid oxide fuel cell applications. *Fuel Processing Technology*. 2015; 140: 21–31. 10.1016/j.fuproc.2015.08.012.

[24] Shah I, Adnan R, Wan Ngah WS, Mohamed N. Iron Impregnated Activated Carbon as an Efficient Adsorbent for the Removal of Methylene Blue: Regeneration and Kinetics Studies. *PLoS ONE*. 2015; 10(4): e0122603 10.1371/journal.pone.0122603. 25849291 PMC4388677

[25] Pokorna D. & Zabranska J. Sulfur-oxidizing bacteria in environmental technology Biotechnology Advances 2015 in press 10.1016/j.biotechadv.2015.02.007. 2011. 25701621

[26] Mescia D, Hernández S.P, Conoci A. & Russo N. MSW landfill biogas desulphurization. *International Journal of Hydrogen Energy*. 2011; 36, 7884–7890.

[27] Choo H. S, Lau L. C, Abdul Rahman M. & Lee K. T. Hydrogen Sulfide adsorption by alkaline impregnated coconut shell activated carbon. *Journal of Engineering Science and Technology*. 2013; 8 (6): 741–753.

[28] Sitthikhankaew R, Predapitakkun S, Kiattikomol R. W, Pumhiran S, Assabumrungrat S. & Laosiripojana N. Comparative study of Hydrogen Sulfide adsorption by using alkaline impregnated activated carbons for hot fuel gas purification. *Energy Procedia*. 2011; 9: 15–24.

[29] Phooratsamee W, Hussaro K, Teekasap S. & Hirunlabh J. Increasing adsorption of activated carbon from Palm Oil Shell for adsorb H_2_S from biogas production by impregnation. *American Journal of Environmental Sciences*. 2014; 10 (5): 431–445.

[30] Mt Yusuf N. Y, Masdar M. S, Isahak W.N.R.W, Nordin D, Husaini T, Majlan E. H, Wu S. Y, Rejab S. A. M. & Lye C. C. Impregnated carbon-ionic liquid as innovative adsorbent for H_2_/CO_2_ separation from biohydrogen. *International Journal of Hydrogen Energy*. 2018.

[31] Sidek M. Z, Jun C. Y, ZULKEFLI N. N, Mt Yusuf N. Y, ISAHAK W. N. R. B. W, Si Tanggang R. & Masdar M. S. Effect of Impregnated Activated Carbon on Carbon Dioxide Adsorption Performance for Biohydrogen Purification. *Materials Research Express*. 2018 10.1088/2053-1591/aae6bf

[32] Zhang L, Chen J, Lv J. X, Wang S. F. & Cui Y. Progress and Development of Capture for CO_2_ by Ionic Liquids—A Review. *Asian J*. *Chem*. 2013; 25 (5): 2355–2358. 10.14233/ajchem.2013.13552.

[33] Nahm S.W, Shim W.G, Park Y. K. & Kim S. C. Thermal and chemical regeneration of spent activated carbon and its adsorption property for toluene. *Chem*. *Eng*. *J*. 2012; 210: 500–509.

[34] Sun K, Jiang J. & Xu J. Chemical regeneration of exhausted granular activated carbon used in citric acid fermentation solution decoloration. Iran. *J*. *Chem*. *Eng*. 2009; 28: 79–83.

[35] Salvador F, Martin-Sanchez N, Sanchez-Hernandez R, Sanchez-Montero M. J. & Izquierdo C. Regeneration of carbonaceous adsorbents. Part I: Thermal Regeneration. *Microporous Mesoporous Mater*. 2015; 202: 259–276.

[36] Salvador F, Martin-Sanchez N, Sanchez-Hernandez R. & Sanchez-Montero M. J. Regeneration of carbonaceous adsorbents. Part II: Chemical, microbiological and vacuum regeneration. *Microporous Mesoporous Mater*. 2015; 202: 277–296.

[37] Sabio E, Gonza ´lez E, Gonza´lez J. F, Gonza´lez Garcı´a C. M, Ramiro A, Gan˜an J. Thermal regeneration of activated carbon saturated with p-nitrophenol. *Carbon*. 2004; 42: 2285–2293.

[38] Conway B. E. & Pell W. G. Power limitations of supercapacitor operation associated with resistance and capacitance distribution in porous electrode devices. *Journal of Power Sources*. 2002; 105 (2): 169.

[39] Isik-Gulsac I. Investigation of impregnated activated carbon properties used in hydrogen sulfide fine removal. *Brazilian Journal of Chemical Engineering*. 2016; 33(4):1021–1030.

[40] Sitthikhankaew R, Chadwick D, Assabumrungrat S. & Laosiripojana N. Effect of KI and KOH impregnations over activated carbon on H_2_S adsorption performance at low and high temperatures. *Separation Science and Technology*. 2014; 49 (3): 354–366.

[41] Okman I, Karagöz A, Tay T. & Erdem M. Activated carbons from grape seeds by chemical activation with Potassium Carbonate and Potassium Hydroxide. *Applied Surface Sci*. 2014; 293: 138–142.

[42] Devil R. S, Selvan C. S. A. & Tamilarasi M. Preparation and characterization of activated carbon from *Caesalpinia Pulcherrima* Pod. *J*. *Environ*. *Nanotechnol*. 2015; 4(2): 19–22.

[43] Yusri A. H, Tan W. L, Noor Hana Hanif A. B. & Mohamad A. B. Surface Characteristics and Catalytic Activity of Copper Deposited Porous Silicon Powder. *Materials*. 2014; 7: 7737–7751. 10.3390/ma7127737 28788272 PMC5456440

[44] Vivekanand G, Ashutosh S. & Nishith V. Removal of SO_2_ by Activated Carbon Fibre Impregnated with Transition Metals. *The Canadian Journal of Chemical Engineering*. 2007; 85.

[45] Gürses A, Ejder-Korucu M. & Doğar Ç. Preparation and characterization of Surfactant-Modified Powder Activated Carbon (SM-PAC) reinforced Poly (Ethylene Oxide) (PEO) composites. *Acta Physica Polonica A*. 2016; 129: 849–852.

[46] Ahamed K. R, Chandrasekaran T. & Arun Kumar A. Characterization of activated carbon prepared from *Albizia Lebbeck* by physical activation. *International Journal of Inter-Disciplinary Research and Innovations*. 2013; 1 (1): 26–31.

[47] Tangjuank S, Insuk N, Udeye V. & Tontrakoon J. Chromium (III) sorption from aqueous solutions using activated carbon prepared from cashew nut shells. *International Journal of Physical Sciences*. 2009; 4 (8): 412–417.

[48] Tan I. A. W, Ahmad A. L. & Hameed B. H. Enhancement of basic dye adsorption uptake from aqueous solutions using chemically modified oil palm shell activated carbon. colloids surfaces A. *Physicochem*. *Eng*. *Aspects*. 2008; 318: 88–96.

[49] Xu H, Yu T, & Li M. Zinc Acetate immobilized on mesoporous materials by acetate ionic liquids as catalysts for vinyl acetate synthesis. *Journal of Chemistry*. 2015.

[50] Vinodhini V. & Das N. Packed bed column studies on Cr (VI) removal from tannery waste water by Neem Sawdust. *Desalination*. 2010; 264: 9–14.

[51] Zulkefli N. N, Masdar M. S, Isahak W.N.R.W, Jahim J, Majlan E. H, Rejab S. A. M. & Lye C. C. Mathematical modelling and simulation on the adsorption of Hydrogen Sulfide (H_2_S) gas. *IOP Conf*. *Ser*: *Mater*. *Sci*. *Eng*. 2017; 206: 012069.

[52] Natalie H. Modeling Hydrogen Sulfide adsorption by activated carbon made from anaerobic digestion by-product. Master Thesis (Department of Chemical Engineering and Applied Chemistry University of Toronto. 2012.

[53] Narbaitz R. M. & Karimi-Jashni A. Electrochemical reactivation of granular activated carbon: Impact of reactor configuration. *Chem*. *Eng*. *J*. 2012; 197: 414–423. 10.1016/j.cej.2012.05.049].

[54] Cui H, Turn S. Q. & Reese M. A. Removal of sulfur compounds from utility pipelined synthetic natural gas using modified activated carbons. *Catalysis Today*. 2008 10.1016/j.cattod.2008.03.024

[55] Soraya H, Iman B, Ehsan M, Farahnaz E.B, Luqman C. A, Thomas S. Y. & Choong. Adsorption of Carbon Dioxide using activated carbon impregnated with Cu promoted by Zinc. *Journal of The Taiwan Institute of Chemical*. 2015: 1–9.

[56] Samanta A, Zhao G. K. H, Shimazu P, Sarkar R. & Gupta. Post-combustion CO_2_ capture using solid sorbents: A Review. *Industrial & Engineering Chemistry Research*. 2012; 51(4): 1438–1463.

[57] Caglayan B. S. & Aksoylu A. E. CO_2_ adsorption on chemically modified activated carbon. *Journal of Hazardous Materials*. 2013; 252: 19–28. 10.1016/j.jhazmat.2013.02.028 23500788

[58] Sreńscek-Nazzal J, Narkiewicz U, Morawski A. W, Wróbel R, Gęsikiewicz-Puchalska A. & Michalkiewicz B. Modification of Commercial activated carbons for CO_2_ adsorption. *Acta Physica Polonica A*. 2016; 129.

[59] Dewil R, Appels L. & Baeyens J. Energy use of biogas hampered by the presence of siloxanes. *Energy Conversion and Management*. 2006; 47:1711–1722.

[60] Farzad S, Taghikhan V, Ghotbi C, Aminshahidi B. & Nemati N. L. Experimental and theoretical study of the effect of moisture on methane adsorption and desorption by activated carbon at 273.3K. *Journal of Natural Gas Chemistry*. 2008; 16: 22–30.

[61] Feng W, Kwon S, Borguet E. & Vidic R. Adsorption of Hydrogen Sulfide onto activatedz carbon fibers: effect of pore structure and surface chemistry. *Environ*. *Sci*. *Technol*. 2005; 39: 9744–9749. 16475362 10.1021/es0507158

