# Supplementary figures and images for: Removal of hydrogen sulfide from a biogas mimic by using impregnated activated carbon adsorbent
Source: PLoS One. 2019 Feb 12;14(2):e0211713. doi: 10.1371/journal.pone.0211713 (PMC6372171; doi:10.1371/journal.pone.0211713)

Electron Image 6

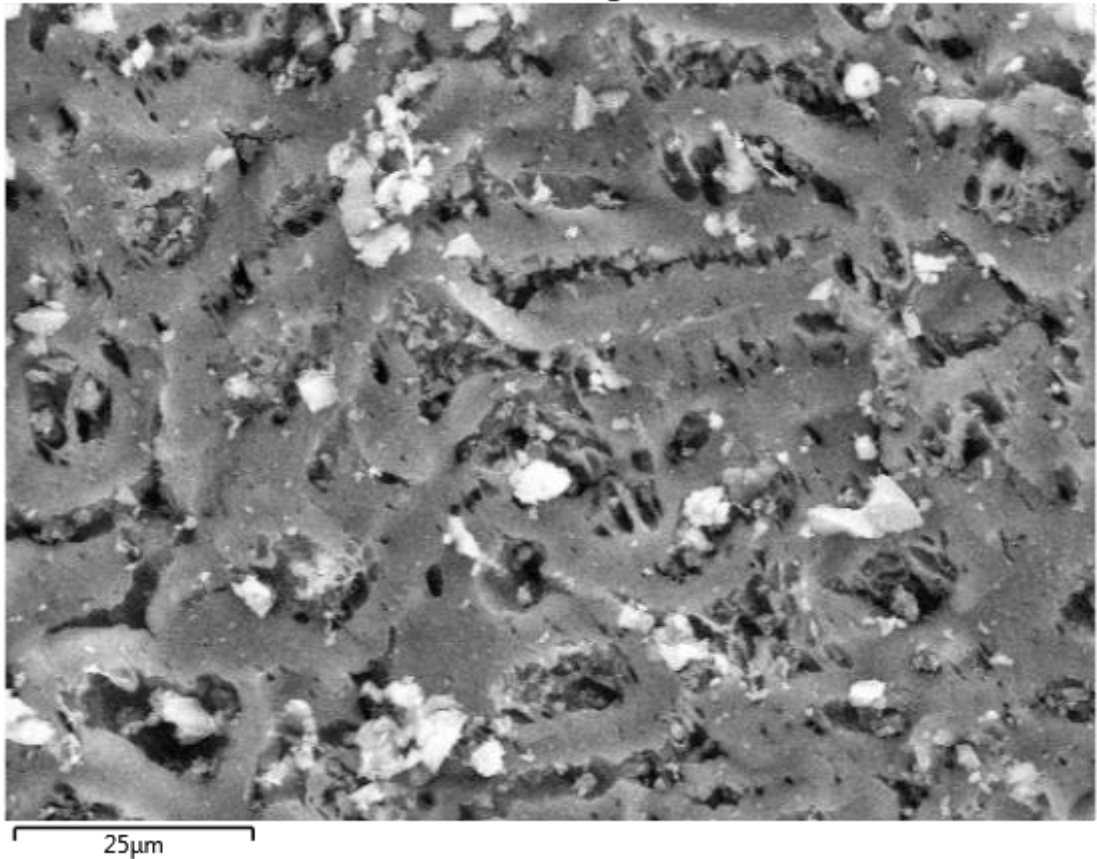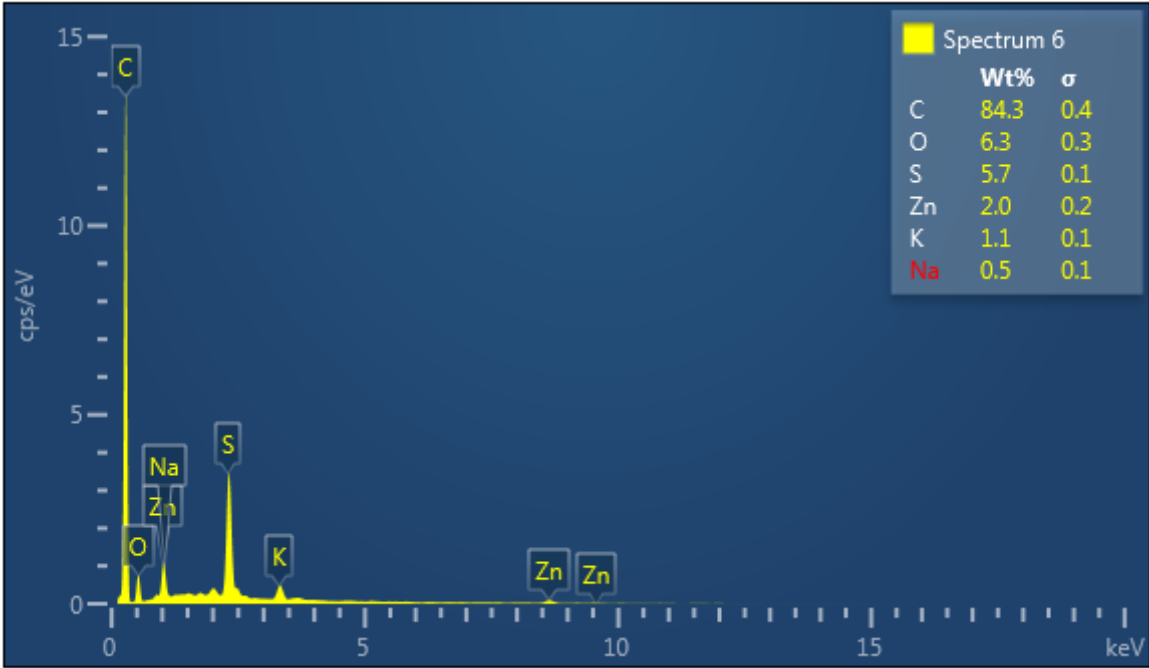

Electron Image 5

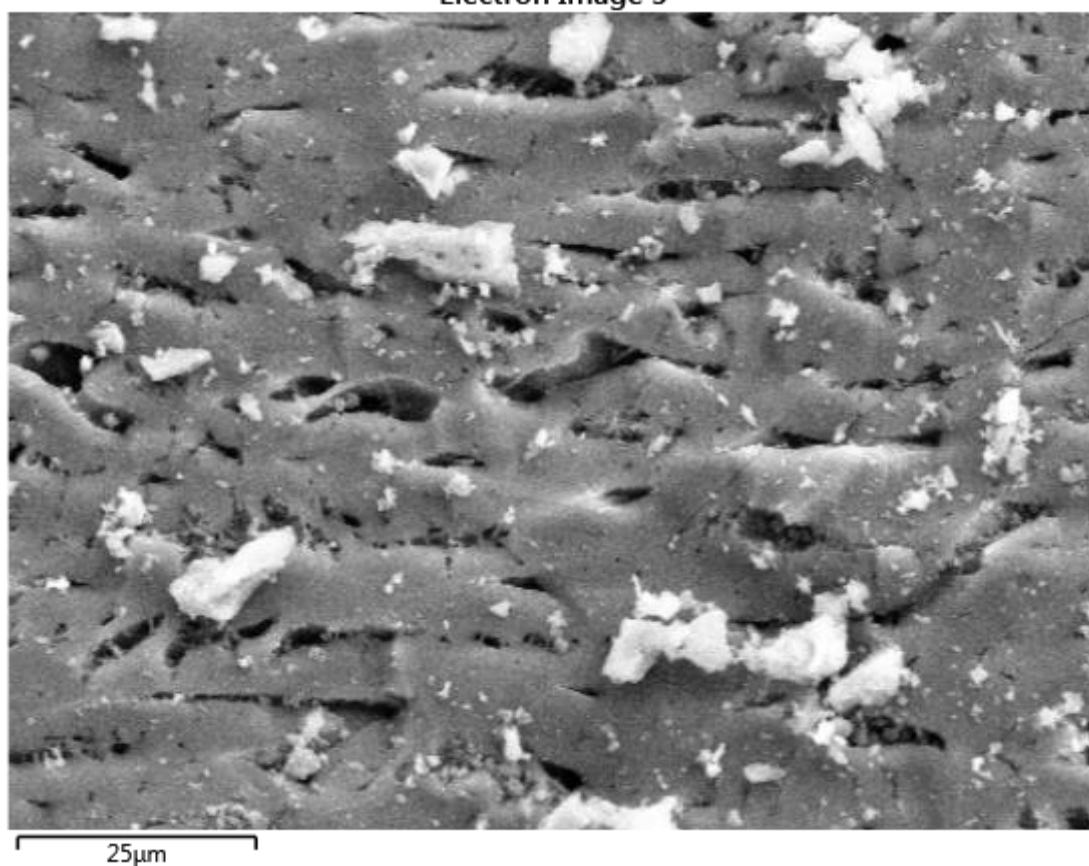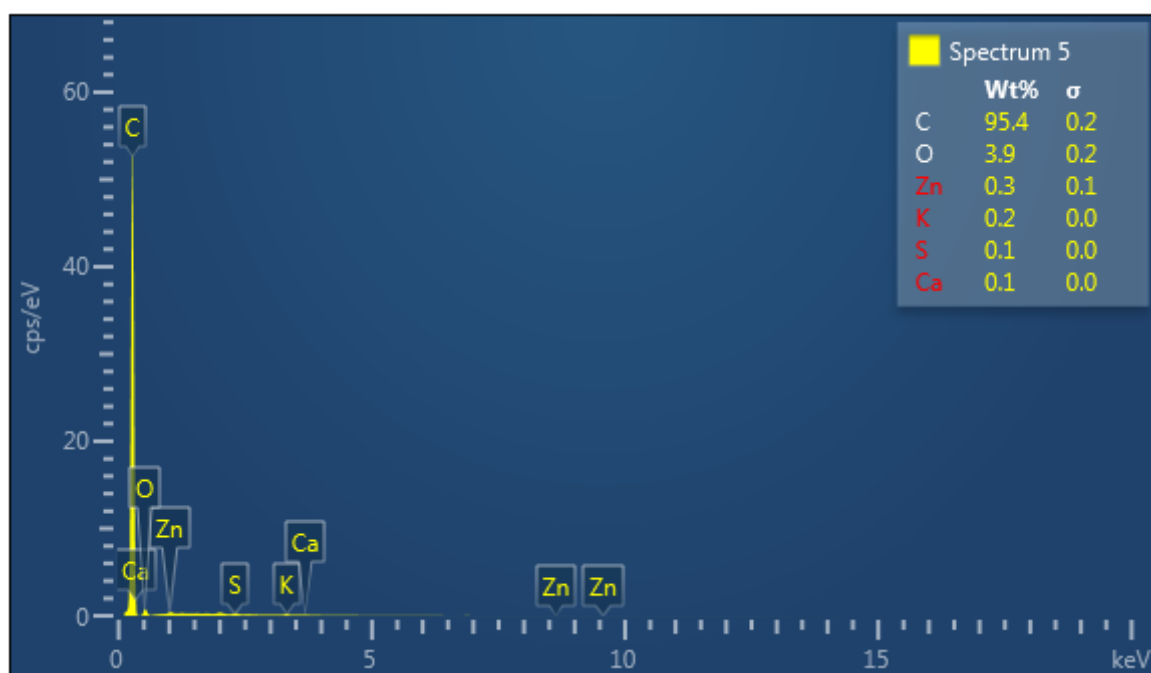

Supplement: S1 Fig — SEM micrograph image of the adsorbents at 2.5 k X (10 μm) (a) ZnAc2–CAC_A; (b) ZnAc2–CAC_D. (PDF) [file pone.0211713.s001.pdf]
