# Supplementary material for: Removal of hydrogen sulfide from a biogas mimic by using impregnated activated carbon adsorbent
Source: PLoS One. 2019 Feb 12;14(2):e0211713. doi: 10.1371/journal.pone.0211713 (PMC6372171; doi:10.1371/journal.pone.0211713)
Supplement: S1 Table — (PDF) [file pone.0211713.s002.pdf]

EDAX TEAM EDS

1_1kx.spc

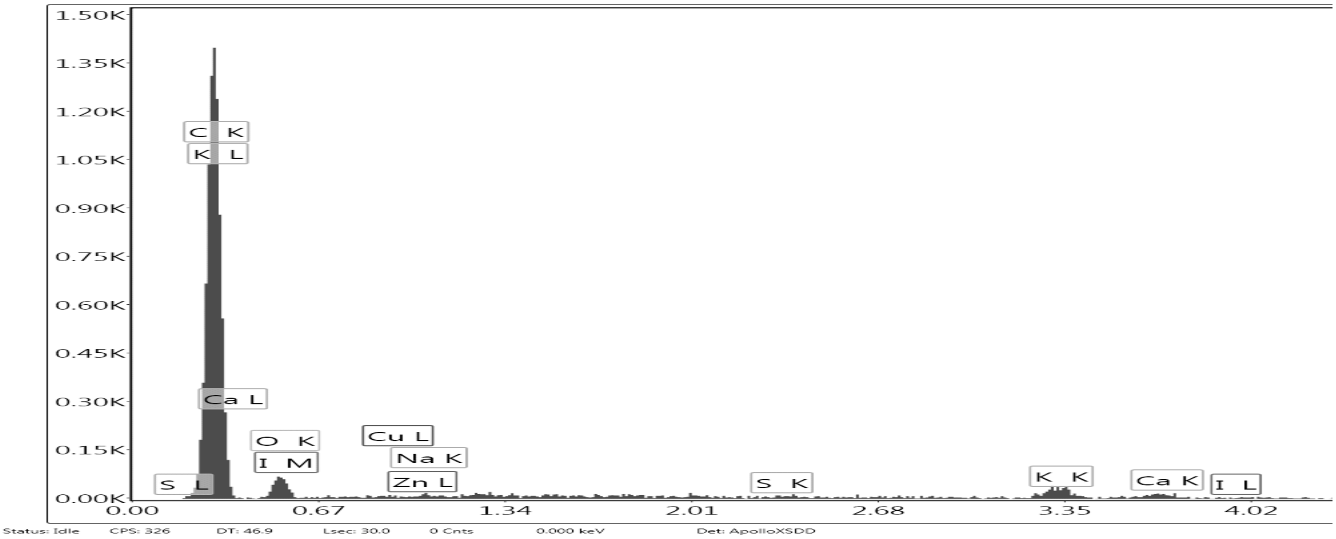

Element	Weight %	Weight %	Atomic %	Error %	Net Intensity	Net Int. Error %	K Ratio	Z	R	A
C K	80.56	80.56	91.36	5.35	212.79	2.05	0.7067	1.0412	0.9801	0.84
O K	5.21	5.21	4.44	35.37	6.79	26.35	0.0141	0.9834	0.9986	0.27
Cu L	0.22	0.22	0.05	86.13	0.52	83.31	0.0014	0.7040	1.1620	0.90
Zn L	0.29	0.29	0.06	78.95	0.71	76.82	0.0020	0.7036	1.1668	0.97
Na K	0.04	0.04	0.02	104.42	0.20	100.00	0.0002	0.8806	1.0198	0.74
S K	1.13	1.13	0.48	27.06	3.84	24.83	0.0098	0.8487	1.0434	0.99
K K	6.24	6.24	2.17	14.02	13.79	11.31	0.0512	0.7956	1.0507	1.00
Ca K	3.19	3.19	1.08	22.56	5.48	19.74	0.0259	0.8070	1.0516	1.00
I L	3.12	3.12	0.33	69.97	1.28	66.43	0.0180	0.5527	1.1775	1.04

EDAX TEAM EDS

2_1kx.spc

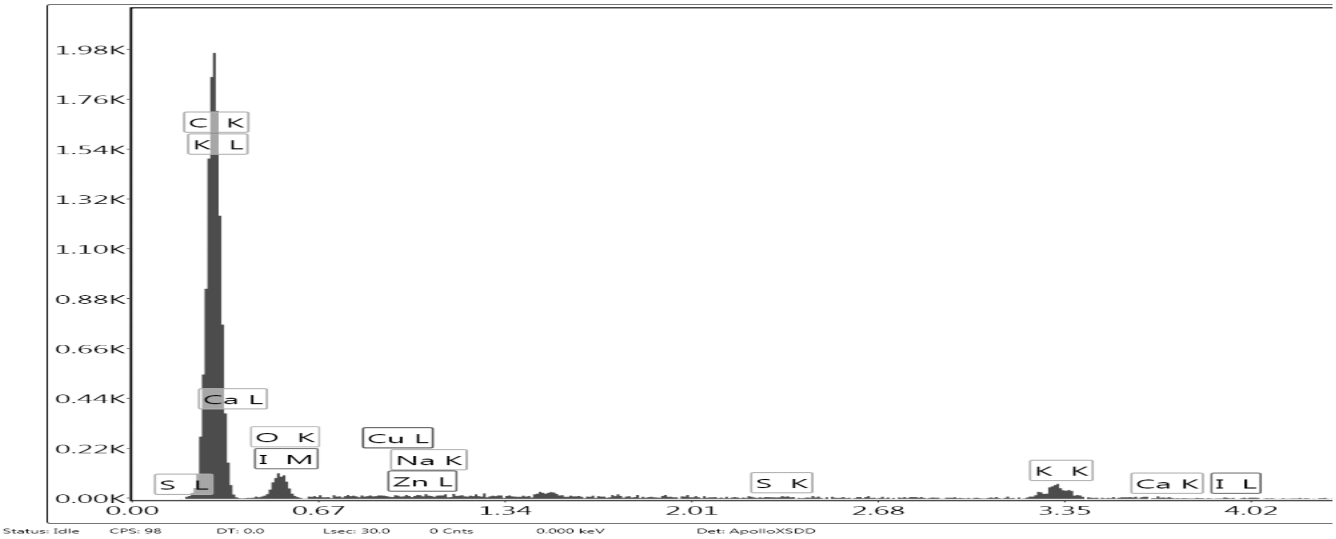

Element	Weight %	Weight %	Atomic %	Error %	Net Intensity	Net Int. Error %	K Ratio	Z	R	A
C K	81.34	81.34	90.24	4.62	310.78	1.55	0.7269	1.0329	0.9847	0.86
O K	7.93	7.93	6.61	22.84	15.03	13.91	0.0219	0.9751	1.0028	0.28
Cu L	0.28	0.28	0.06	75.48	0.96	72.75	0.0018	0.6979	1.1662	0.91
Zn L	0.42	0.42	0.09	69.05	1.49	67.02	0.0029	0.6974	1.1709	0.98
Na K	0.00	0.00	0.00	104.35	0.03	100.00	0.0000	0.8728	1.0234	0.75
S K	0.71	0.71	0.29	33.22	3.38	31.00	0.0061	0.8409	1.0460	0.99
K K	6.81	6.81	2.32	12.76	20.88	10.04	0.0545	0.7881	1.0528	1.01
Ca K	0.58	0.58	0.19	77.13	1.39	74.30	0.0046	0.7993	1.0535	1.00
I L	1.93	1.93	0.20	69.72	1.11	66.16	0.0110	0.5473	1.1793	1.04

EDAX TEAM EDS

3_1kx.spc

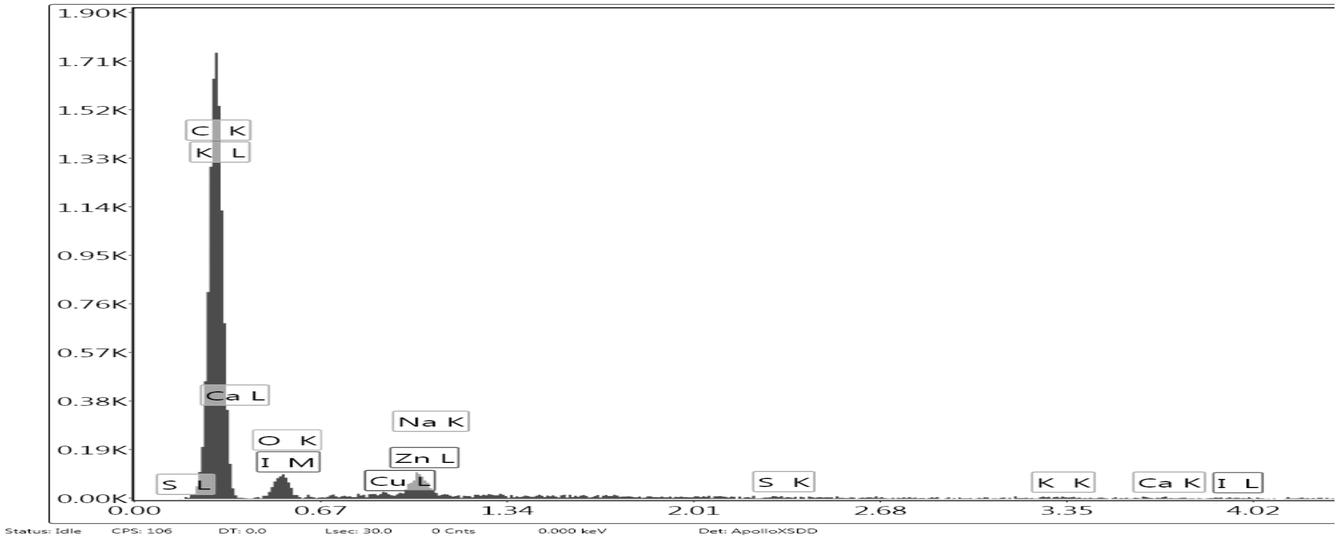

Element	Weight %	Weight %	Atomic %	Error %	Net Intensity	Net Int. Error %	K Ratio	Z	R	A
C K	90.18	90.18	95.84	5.50	262.69	2.26	0.7810	1.0214	0.9893	0.84
O K	3.62	3.62	2.89	60.97	5.38	52.07	0.0100	0.9639	1.0069	0.28
Cu L	0.08	0.08	0.02	102.22	0.23	100.00	0.0005	0.6897	1.1703	0.96
Zn L	4.37	4.37	0.85	14.90	12.45	12.78	0.0308	0.6892	1.1749	1.02
Na K	0.00	0.00	0.00	104.18	0.03	100.00	0.0000	0.8626	1.0269	0.76
S K	0.21	0.21	0.08	86.25	0.77	84.02	0.0018	0.8308	1.0486	0.99
K K	0.47	0.47	0.15	76.18	1.13	73.46	0.0037	0.7785	1.0548	1.01
Ca K	0.25	0.25	0.08	96.86	0.47	93.96	0.0020	0.7896	1.0553	1.01
I L	0.81	0.81	0.08	89.93	0.37	86.32	0.0046	0.5406	1.1810	1.05

EDAX TEAM EDS

4_1kx.spc

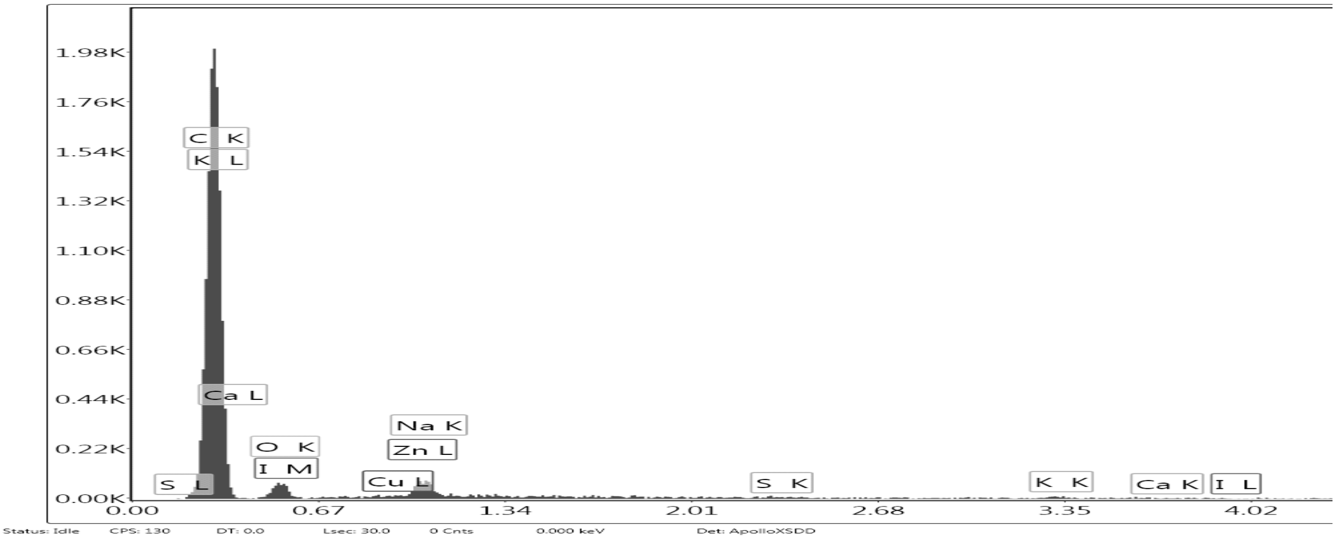

Element	Weight %	Weight %	Atomic %	Error %	Net Intensity	Net Int. Error %	K Ratio	Z	R	A
C K	90.69	90.69	94.22	4.18	316.63	1.49	0.8285	1.0113	0.9954	0.90
O K	5.52	5.52	4.30	29.25	9.28	20.36	0.0152	0.9539	1.0122	0.28
Cu L	0.00	0.00	0.00	102.25	0.01	100.00	0.0000	0.6822	1.1757	0.95
Zn L	0.39	0.39	0.07	81.08	1.25	78.90	0.0027	0.6817	1.1802	1.03
Na K	1.78	1.78	0.97	15.11	12.89	11.13	0.0120	0.8531	1.0314	0.78
S K	0.31	0.31	0.12	71.94	1.27	69.67	0.0026	0.8211	1.0518	1.00
K K	0.69	0.69	0.22	68.62	1.84	65.88	0.0054	0.7692	1.0574	1.01
Ca K	0.16	0.16	0.05	96.18	0.34	93.26	0.0012	0.7800	1.0576	1.01
I L	0.46	0.46	0.05	92.46	0.23	88.83	0.0026	0.5340	1.1832	1.05

EDAX TEAM EDS

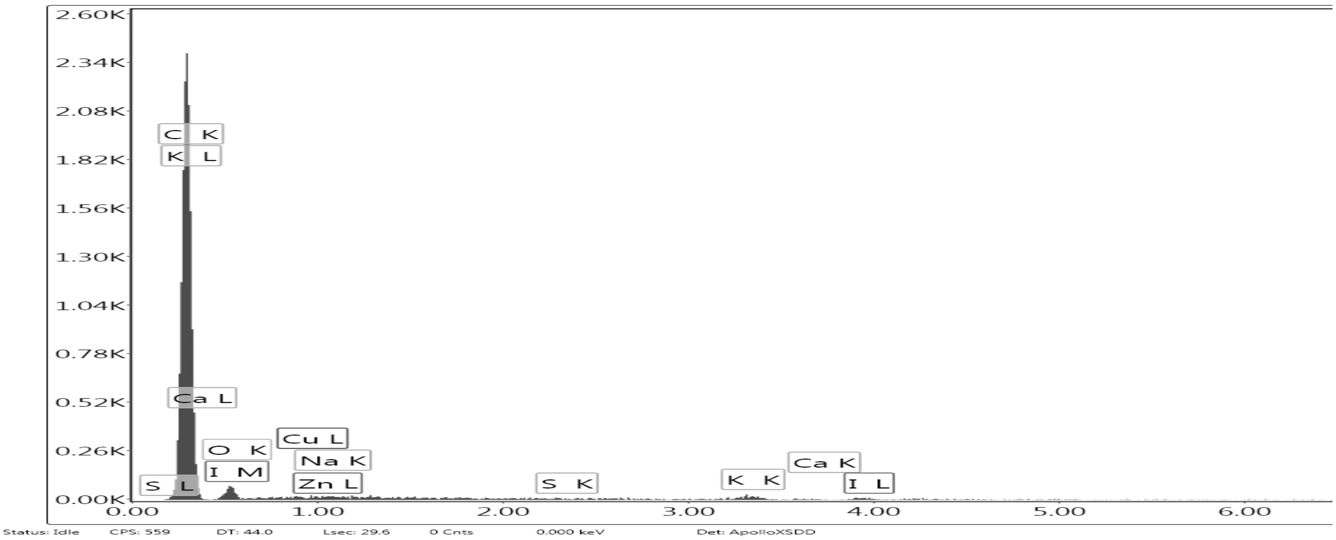

Element	Weight %	Weight %	Atomic %	Error %	Net Intensity	Net Int. Error %	K Ratio	Z	R	A
C K	84.72	84.72	96.64	5.02	367.33	1.83	0.7757	1.0544	0.9729	0.86
O K	0.43	0.43	0.37	108.70	1.00	100.00	0.0014	0.9950	0.9920	0.32
Cu L	0.52	0.52	0.11	41.16	1.92	38.26	0.0034	0.7080	1.1533	0.91
Zn L	0.76	0.76	0.16	29.83	2.84	27.43	0.0052	0.7075	1.1581	0.96
Na K	0.00	0.00	0.00	104.50	0.03	100.00	0.0000	0.8904	1.0138	0.75
S K	1.02	1.02	0.43	24.31	4.77	21.75	0.0087	0.8573	1.0379	0.99
K K	3.41	3.41	1.19	16.44	9.97	13.40	0.0279	0.8029	1.0449	1.00
Ca K	0.44	0.44	0.15	83.35	0.99	80.09	0.0036	0.8139	1.0454	1.00
I L	8.70	8.70	0.94	30.67	4.54	26.65	0.0497	0.5493	1.1568	1.04

EDAX TEAM EDS

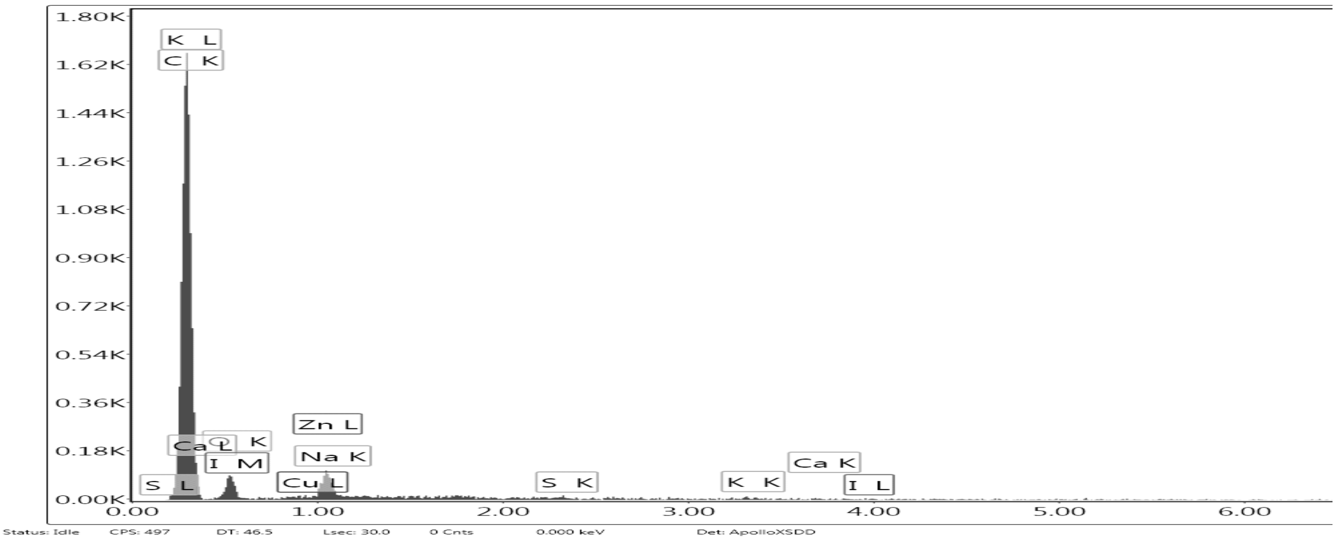

Element	Weight %	Weight %	Atomic %	Error %	Net Intensity	Net Int. Error %	K Ratio	Z	R	A
C K	88.27	88.27	92.44	4.71	254.81	1.78	0.7870	1.0133	0.9949	0.87
O K	6.95	6.95	5.46	30.14	10.14	21.31	0.0196	0.9558	1.0118	0.29
Cu L	0.04	0.04	0.01	102.29	0.10	100.00	0.0002	0.6836	1.1753	0.95
Zn L	0.02	0.02	0.00	102.14	0.04	100.00	0.0001	0.6830	1.1798	1.02
Na K	2.91	2.91	1.59	12.72	17.79	8.73	0.0196	0.8549	1.0311	0.78
S K	0.42	0.42	0.17	71.13	1.48	68.87	0.0035	0.8228	1.0516	1.00
K K	0.71	0.71	0.23	70.45	1.60	67.72	0.0055	0.7707	1.0572	1.01
Ca K	0.17	0.17	0.05	102.92	0.31	100.00	0.0014	0.7816	1.0575	1.01
I L	0.52	0.52	0.05	96.09	0.22	92.47	0.0029	0.5350	1.1831	1.05
