# Supplementary material for: Removal of hydrogen sulfide from a biogas mimic by using impregnated activated carbon adsorbent
Source: PLoS One. 2019 Feb 12;14(2):e0211713. doi: 10.1371/journal.pone.0211713 (PMC6372171; doi:10.1371/journal.pone.0211713)
Supplement: S2 Table — (PDF) [file pone.0211713.s003.pdf]

Sample:	001-625-KI-CAC		
Operator:	Jack		
Submitter:	Am		
File:	C:\2020\DATA_INI\001-625.SMP		

Started:	25-011-17 6:27:49PM	Analysis Adsorptive:	N2
Completed:	26-011-17 6:55:33AM	Analysis Bath Temp.:	-195.704 °C
Report Time:	01-022-17 6:03:56PM	Thermal Correction:	No
Sample Mass:	0.1910 g	Warm Free Space:	27.6671 cm³ Measured
Cold Free Space:	83.7949 cm³	Equilibration Interval:	10 s
Ambient Temperature:	22.00 °C	Low Pressure Dose:	5.000 cm³/g STP
Automatic Degas:	Yes		

Summary Report

Surface Area

Single point surface area at P/Po = 0.201845721:	748.2817 m²/g
BET Surface Area:	726.6914 m²/g
Langmuir Surface Area:	967.0150 m²/g
t-Plot Micropore Area:	574.8799 m²/g
t-Plot External Surface Area:	151.8115 m²/g

BJH Adsorption cumulative surface area of pores between 17.000 Å and 3000.000 Å width:	64.712 m^2/g
BJH Desorption cumulative surface area of pores between 17.000 Å and 3000.000 Å width:	68.3421 m^2/g

Pore Volume

Single point adsorption total pore volume of pores less than 1249.773 Å width at P/Po = 0.984263997:	0.349330 cm^3/g
Single point desorption total pore volume of pores less than 730.795 Å width at P/Po = 0.972785932:	0.348985 cm^3/g
t-Plot micropore volume:	0.266402 cm^3/g
BJH Adsorption cumulative volume of pores between 17.000 Å and 3000.000 Å width:	0.047780 cm^3/g
BJH Desorption cumulative volume of pores between 17.000 Å and 3000.000 Å width:	0.047319 cm^3/g

Pore Size

Adsorption average pore width (4V/A by BET):	19.2285 Å
Desorption average pore width (4V/A by BET):	19.2095 Å
BJH Adsorption average pore width (4V/A):	29.534 Å
BJH Desorption average pore width (4V/A):	27.695 Å

Sample:	001-626- KOH-CAC		
Operator:	Jack		
Submitter:	Am		
File:	C:\2020\DATA_INI\001-626.SMP		
Started:	26-011-17 9:43:35AM	Analysis Adsorptive:	N2
Completed:	27-011-17 12:33:09AM	Analysis Bath Temp.:	-195.683 °C
Report Time:	01-022-17 6:03:59PM	Thermal Correction:	No
Sample Mass:	0.1761 g	Warm Free Space:	25.0765 cm³ Measured
Cold Free Space:	64.2053 cm³	Equilibration Interval:	10 s
Ambient Temperature:	22.00 °C	Low Pressure Dose:	5.000 cm³/g STP
Automatic Degas:	Yes		

Summary Report

Surface Area

Single point surface area at P/Po = 0.202600453:	825.8336 m²/g
BET Surface Area:	805.4541 m²/g
Langmuir Surface Area:	1073.5670 m²/g
t-Plot Micropore Area:	613.7734 m²/g
t-Plot External Surface Area:	191.6807 m²/g

BJH Adsorption cumulative surface area of pores between 17.000 Å and 3000.000 Å width:	68.476 m²/g
BJH Desorption cumulative surface area of pores between 17.000 Å and 3000.000 Å width:	82.3445 m²/g

Pore Volume

Single point adsorption total pore volume of pores less than 0.000 Å width at P/Po = 1.003623644:	0.367052 cm³/g
Single point desorption total pore volume of pores less than 631.082 Å width at P/Po = 0.968372929:	0.368099 cm³/g
t-Plot micropore volume:	0.283633 cm³/g
BJH Adsorption cumulative volume of pores between 17.000 Å and 3000.000 Å width:	0.039114 cm³/g
BJH Desorption cumulative volume of pores between 17.000 Å and 3000.000 Å width:	0.047337 cm³/g

Pore Size

Adsorption average pore width (4V/A by BET):	18.2283 Å
Desorption average pore width (4V/A by BET):	18.2803 Å
BJH Adsorption average pore width (4V/A):	22.848 Å
BJH Desorption average pore width (4V/A):	22.995 Å

Sample:	001-627-RAW CAC
Operator:	Jack
Submitter:	Am
File:	C:\2020\DATA_INI\001-627.SMP

Started:	27-011-17 4:31:56AM	Analysis Adsorptive:	N2
Completed:	27-011-17 2:50:56PM	Analysis Bath Temp.:	-195.696 °C
Report Time:	01-022-17 6:04:03PM	Thermal Correction:	No
Sample Mass:	0.0571 g	Warm Free Space:	27.5183 cm³ Measured
Cold Free Space:	81.7763 cm³	Equilibration Interval:	10 s
Ambient Temperature:	22.00 °C	Low Pressure Dose:	5.000 cm³/g STP
Automatic Degas:	Yes		

Summary Report

Surface Area

Single point surface area at P/Po = 0.200817843:	923.5674 m²/g
BET Surface Area:	901.0417 m²/g
Langmuir Surface Area:	1196.0140 m²/g
t-Plot Micropore Area:	693.6494 m²/g
t-Plot External Surface Area:	207.3922 m²/g

BJH Adsorption cumulative surface area of pores between 17.000 Å and 3000.000 Å width:	87.437 m^2/g
BJH Desorption cumulative surface area of pores between 17.000 Å and 3000.000 Å width:	103.4942 m^2/g

Pore Volume

Single point adsorption total pore volume of pores less than 1283.897 Å width at P/Po = 0.984689712:	0.430543 cm^3/g
Single point desorption total pore volume of pores less than 1096.822 Å width at P/Po = 0.982025460:	0.431053 cm^3/g
t-Plot micropore volume:	0.319614 cm^3/g
BJH Adsorption cumulative volume of pores between 17.000 Å and 3000.000 Å width:	0.063219 cm^3/g
BJH Desorption cumulative volume of pores between 17.000 Å and 3000.000 Å width:	0.072732 cm^3/g

Pore Size

Adsorption average pore width (4V/A by BET):	19.1131 Å
Desorption average pore width (4V/A by BET):	19.1358 Å
BJH Adsorption average pore width (4V/A):	28.921 Å
BJH Desorption average pore width (4V/A):	28.111 Å

Sample:	001-628- $ZnAC_2$-CAC
Operator:	Jack
Submitter:	Am
File:	C:\2020\DATA_INI\001-628.SMP

Started:	30-011-17 9:40:52AM	Analysis Adsorptive:	N2
Completed:	30-011-17 9:19:36PM	Analysis Bath Temp.:	-195.712 °C
Report Time:	01-022-17 6:04:06PM	Thermal Correction:	No
Sample Mass:	0.0687 g	Warm Free Space:	25.1026 cm³ Measured
Cold Free Space:	64.5609 cm³	Equilibration Interval:	10 s
Ambient Temperature:	22.00 °C	Low Pressure Dose:	5.000 cm³/g STP
Automatic Degas:	Yes		

Summary Report

Surface Area

Single point surface area at P/Po = 0.200893070:	672.1401 m²/g
BET Surface Area:	656.7483 m²/g
Langmuir Surface Area:	872.8989 m²/g
t-Plot Micropore Area:	500.5086 m²/g
t-Plot External Surface Area:	156.2397 m²/g

BJH Adsorption cumulative surface area of pores between 17.000 Å and 3000.000 Å width:	54.067 m²/g
BJH Desorption cumulative surface area of pores between 17.000 Å and 3000.000 Å width:	0.6153 m²/g

Pore Volume

Single point adsorption total pore volume of pores less than 1399.805 Å width at P/Po = 0.985978762:	0.294462 cm³/g
Single point desorption total pore volume of pores less than 903.160 Å width at P/Po = 0.978084395:	0.296155 cm³/g
t-Plot micropore volume:	0.230162 cm³/g
BJH Adsorption cumulative volume of pores between 17.000 Å and 3000.000 Å width:	0.041467 cm³/g
BJH Desorption cumulative volume of pores between 17.000 Å and 3000.000 Å width:	0.020279 cm³/g

Pore Size

Adsorption average pore width (4V/A by BET):	17.9345 Å
Desorption average pore width (4V/A by BET):	18.0377 Å
BJH Adsorption average pore width (4V/A):	30.678 Å
BJH Desorption average pore width (4V/A):	1318.213 Å

Sample:	001-629-$CuSO_4$-CAC
Operator:	Jack
Submitter:	Am
File:	C:\2020\DATA_INI\001-629.SMP

Started:	30-011-17 10:06:25PM	Analysis Adsorptive:	N2
Completed:	31-011-17 10:40:13AM	Analysis Bath Temp.:	-195.712 °C
Report Time:	01-022-17 6:04:09PM	Thermal Correction:	No
Sample Mass:	0.0404 g	Warm Free Space:	27.7241 cm³ Measured
Cold Free Space:	79.0580 cm³	Equilibration Interval:	10 s
Ambient Temperature:	22.00 °C	Low Pressure Dose:	5.000 cm³/g STP
Automatic Degas:	Yes		

Summary Report

Surface Area

Single point surface area at P/Po = 0.300693605:	839.0978 m²/g
BET Surface Area:	39.7642 m²/g
Langmuir Surface Area:	1211.7604 m²/g
t-Plot External Surface Area:	208.0819 m²/g
BJH Adsorption cumulative surface area of pores between 17.000 Å and 3000.000 Å width:	140.709 m²/g

Pore Volume

Single point adsorption total pore volume of pores less than 3873.040 Å width at P/Po = 0.995000000:	0.433569 cm³/g
Single point desorption total pore volume of pores less than 3873.040 Å width at P/Po = 0.995000000:	0.433569 cm³/g
t-Plot micropore volume:	0.329269 cm³/g
BJH Adsorption cumulative volume of pores between 17.000 Å and 3000.000 Å width:	0.075049 cm³/g

Pore Size

Adsorption average pore width (4V/A by BET):	436.1405 Å
Desorption average pore width (4V/A by BET):	436.1405 Å
BJH Adsorption average pore width (4V/A):	21.335 Å

DFT Pore Size

Volume in Pores	<	14.83 Å	:	0.25456 cm³/g
Total Volume in Pores	<=	147.61 Å	:	0.60436 cm³/g
Area in Pores	>	147.61 Å	:	0.000 m²/g
Total Area in Pores	>=	14.83 Å	:	109.307 m²/g

DFT Surface Energy

Total Area : 1274.233 m²/g

Horvath-Kawazoe

Maximum pore volume at P/Po = 0.551530618:	0.429742 cm³/g

Sample:	001-630-Na_2CO_3-CAC
Operator:	Jack
Submitter:	Am
File:	C:\2020\DATA_INI\001-630.SMP

Started:	31-011-17 11:05:19AM	Analysis Adsorptive:	N2
Completed:	31-011-17 10:28:05PM	Analysis Bath Temp.:	-195.729 °C
Report Time:	01-022-17 6:04:12PM	Thermal Correction:	No
Sample Mass:	0.0941 g	Warm Free Space:	25.2006 cm³ Measured
Cold Free Space:	73.6520 cm³	Equilibration Interval:	10 s
Ambient Temperature:	22.00 °C	Low Pressure Dose:	5.000 cm³/g STP
Automatic Degas:	Yes		

Summary Report

Surface Area

Single point surface area at P/Po = 0.201216119:	918.6860 m²/g
BET Surface Area:	901.5779 m²/g
Langmuir Surface Area:	1195.6562 m²/g
t-Plot Micropore Area:	666.1636 m²/g
t-Plot External Surface Area:	235.4143 m²/g

BJH Adsorption cumulative surface area of pores between 17.000 Å and 3000.000 Å width:	94.463 m²/g
BJH Desorption cumulative surface area of pores between 17.000 Å and 3000.000 Å width:	100.9962 m²/g

Pore Volume

Single point adsorption total pore volume of pores less than 1273.238 Å width at P/Po = 0.984559216:	0.428111 cm³/g
Single point desorption total pore volume of pores less than 789.692 Å width at P/Po = 0.974860916:	0.427786 cm³/g
t-Plot micropore volume:	0.305281 cm³/g
BJH Adsorption cumulative volume of pores between 17.000 Å and 3000.000 Å width:	0.064933 cm³/g
BJH Desorption cumulative volume of pores between 17.000 Å and 3000.000 Å width:	0.066433 cm³/g

Pore Size

Adsorption average pore width (4V/A by BET):	18.9939 Å
Desorption average pore width (4V/A by BET):	18.9794 Å
BJH Adsorption average pore width (4V/A):	27.496 Å
BJH Desorption average pore width (4V/A):	26.311 Å
