# Supplementary material for: Removal of hydrogen sulfide from a biogas mimic by using impregnated activated carbon adsorbent
Source: PLoS One. 2019 Feb 12;14(2):e0211713. doi: 10.1371/journal.pone.0211713 (PMC6372171; doi:10.1371/journal.pone.0211713)
Supplement: S3 Table — (PDF) [file pone.0211713.s004.pdf]

Raw Commercial Activated Carbon (coconut)

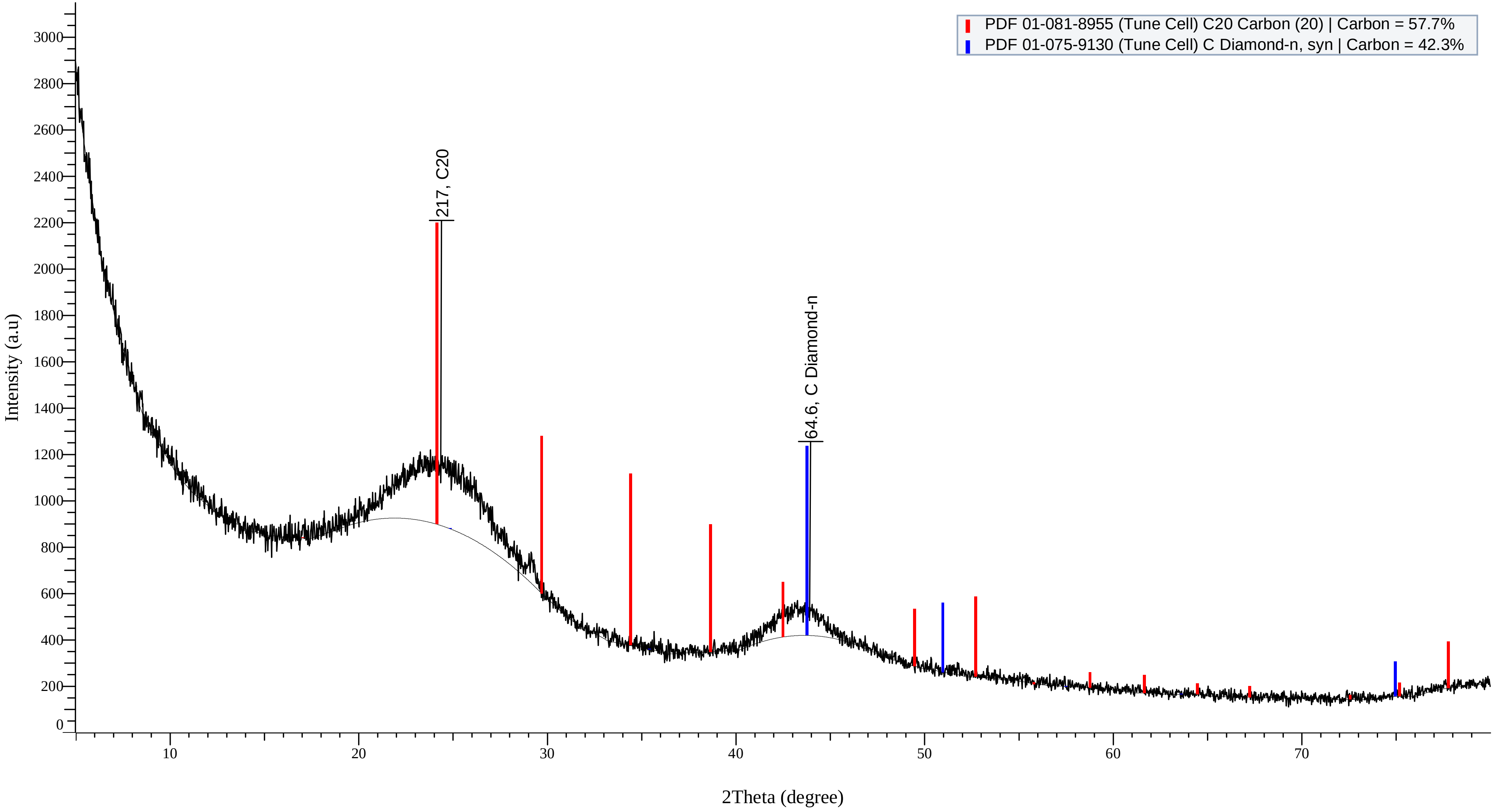

Raw CAC Overall Data

**Visible**	Yes
**Icon**	
**Color**	
**Index**	1
**Name**	Raw CAC.raw
**Parent**	2Theta
**Sample Name**	Raw CAC
**Scan's Sample Name**	
**File Name**	Raw CAC.raw
**Experiment File Name**	
**Database ID**	
**Scan Type**	Coupled TwoTheta/Theta
**Scan Mode**	Continuousscan
**Scan Status**	Completed
**Start**	5.000
**End**	79.986
**Step Size**	0.025
**Time per Step**	19.00
**Axis Offset**	
**Temperature**	25 °C (Room)
**Temp. Diff.**	
**Heating Rate**	
**Time Started**	15.0
**Goniometer Radius**	217.5
**Goniometer Radius Primary**	
**Goniometer Radius Secondary**	
**2-theta**	5.000
**2-theta Osc. Amplitude**	0.000
**2-theta Osc. Speed**	0.000
**Theta**	2.500
**Theta Osc. Amplitude**	0.000
**Theta Osc. Speed**	0.000
**Detector**	
**Detector Osc. Amplitude**	
**Detector Osc. Speed**	
**Chi**	0.00
**Chi Osc. Amplitude**	0.00
**Chi Osc. Speed**	0.00
**Phi**	0.00
**Phi Osc. Amplitude**	0.00

**Phi Osc. Speed**	0.00
**Gamma**	0.00
**X-Drive**	0.0
**X-drive Osc. Amplitude**	0.0
**X-drive Osc. Speed**	0.0
**Y-Drive**	0.0
**Y-drive Osc. Amplitude**	0.0
**Y-drive Osc. Speed**	0.0
**Z-Drive**	0.0
**Z-drive Osc. Amplitude**	0.0
**Z-drive Osc. Speed**	0.0
**Aux1**	0.0
**Aux2**	0.0
**Aux3**	0.0
**Anode**	Cu
**kα1**	1.54060
**kα2**	1.54439
**kα2 Ratio**	0.50000
**kβ**	1.39222
**Generator kV**	40.0
**Generator mA**	40.0
**Detector Name**	Braun
**Calcium Channel**	
**Calcium Channel Time**	
**LynxEye 0D**	No
**PSD Opening**	
**PSD Opening Initial**	
**2Theta For Final PSD Opening**	
**Detector Slit Opening**	12.930
**Lower Discriminator**	
**Upper Discriminator**	
**Discriminator Width**	
**Detector Counts 0D**	
**Primary Soller slit**	4.000
**Secondary Soller slit**	4.000
**Center Soller slit**	
**Sample rotation speed**	
**Divergence Slit**	0.298
**Antiscatter Slit**	0.298
**Slit Mode**	Fixed
**Simul. Slit Mode**	
**Center Slit**	

**X-Offset**	0.000
**Displacement**	0.000
**Y-Scale Factor**	1
**Y-Offset**	0
**Humidity**	n.a.
**Curvature**	1.000
**Threshold**	1.000
**Enhanced**	No
**BackgroundFit**	No
**BackgroundOrder**	0.000
**BackgroundHyperbola**	No
**Compute Crystallinity**	Yes
**Cristallinity - From**	5.000
**Cristallinity - To**	79.986
**%-Crystallinity**	37.8%
**%-Amorphous**	62.2%
**Global Area**	324.4
**Reduced Area**	122.8
**Transmission without Blank**	
**Transmission**	
**Company Name**	Malaysia
**Operator Name**	Administrator
**User Comment**	
**Sample Position**	
**Creation Date/Time**	4/1/2017 3:40:41 PM
**Last Write Date/Time**	4/1/2017 3:40:41 PM
**Measurement Duration**	00:00:00
**Set index**	Not used

Raw CAtC Compute Crystallinity

**Index**	1
**Sample Name**	Raw CAC
**%-Crystallinity**	37.8%
**%-Amorphous**	62.2%
**Global Area**	324.4
**Reduced Area**	122.8

Modified Na2CO3 - CAC

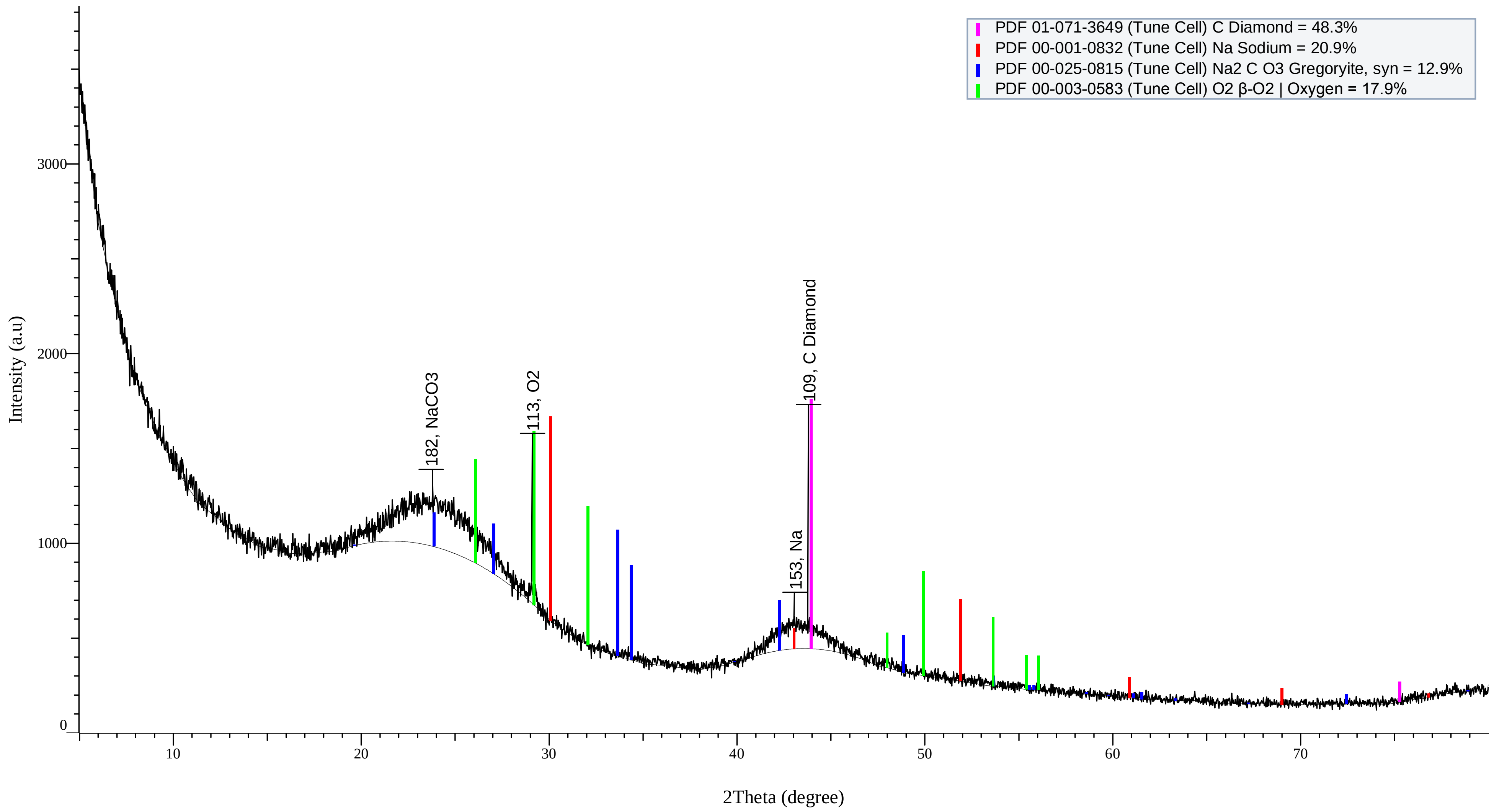

Na2CO3-CAC Overall Data

**Visible**	Yes
**Icon**	
**Color**	
**Index**	1
**Name**	CAC-Na2CO3.raw
**Parent**	2Theta
**Sample Name**	CAC-Na2CO3
**Scan's Sample Name**	
**File Name**	CAC-Na2CO3.raw
**Experiment File Name**	
**Database ID**	
**Scan Type**	Coupled TwoTheta/Theta
**Scan Mode**	Continuousscan
**Scan Status**	Completed
**Start**	5.000
**End**	79.986
**Step Size**	0.025
**Time per Step**	19.00
**Axis Offset**	
**Temperature**	25 °C (Room)
**Temp. Diff.**	
**Heating Rate**	
**Time Started**	17.0
**Goniometer Radius**	217.5
**Goniometer Radius Primary**	
**Goniometer Radius Secondary**	
**2-theta**	5.000
**2-theta Osc. Amplitude**	0.000
**2-theta Osc. Speed**	0.000
**Theta**	2.500
**Theta Osc. Amplitude**	0.000
**Theta Osc. Speed**	0.000
**Detector**	
**Detector Osc. Amplitude**	
**Detector Osc. Speed**	
**Chi**	0.00
**Chi Osc. Amplitude**	0.00
**Chi Osc. Speed**	0.00
**Phi**	0.00
**Phi Osc. Amplitude**	0.00

**Phi Osc. Speed**	0.00
**Gamma**	0.00
**X-Drive**	0.0
**X-drive Osc. Amplitude**	0.0
**X-drive Osc. Speed**	0.0
**Y-Drive**	0.0
**Y-drive Osc. Amplitude**	0.0
**Y-drive Osc. Speed**	0.0
**Z-Drive**	0.0
**Z-drive Osc. Amplitude**	0.0
**Z-drive Osc. Speed**	0.0
**Aux1**	0.0
**Aux2**	0.0
**Aux3**	0.0
**Anode**	Cu
**kα1**	1.54060
**kα2**	1.54439
**kα2 Ratio**	0.50000
**kβ**	1.39222
**Generator kV**	40.0
**Generator mA**	40.0
**Detector Name**	Braun
**Calcium Channel**	
**Calcium Channel Time**	
**LynxEye 0D**	No
**PSD Opening**	
**PSD Opening Initial**	
**2Theta For Final PSD Opening**	
**Detector Slit Opening**	12.930
**Lower Discriminator**	
**Upper Discriminator**	
**Discriminator Width**	
**Detector Counts 0D**	
**Primary Soller slit**	4.000
**Secondary Soller slit**	4.000
**Center Soller slit**	
**Sample rotation speed**	
**Divergence Slit**	0.298
**Antiscatter Slit**	0.298
**Slit Mode**	Fixed
**Simul. Slit Mode**	
**Center Slit**	

**X-Offset**	0.000
**Displacement**	0.000
**Y-Scale Factor**	1
**Y-Offset**	0
**Humidity**	n.a.
**Curvature**	1.000
**Threshold**	1.000
**Enhanced**	No
**BackgroundFit**	No
**BackgroundOrder**	0.000
**BackgroundHyperbola**	No
**Compute Crystallinity**	Yes
**Cristallinity - From**	5.000
**Cristallinity - To**	79.986
**%-Crystallinity**	37.0%
**%-Amorphous**	63.0%
**Global Area**	331.5
**Reduced Area**	122.8
**Transmission without Blank**	
**Transmission**	
**Company Name**	Malaysia
**Operator Name**	Administrator
**User Comment**	
**Sample Position**	
**Creation Date/Time**	4/1/2017 3:35:01 PM
**Last Write Date/Time**	4/1/2017 3:35:01 PM
**Measurement Duration**	00:00:00
**Set index**	Not used

Na2CO3- CAC Compute Crystallinity

**Index**	1
**Sample Name**	CAC-Na2CO3
**%-Crystallinity**	37.0%
**%-Amorphous**	63.0%
**Global Area**	331.5
**Reduced Area**	122.8

Modified ZnAc2 - CAC

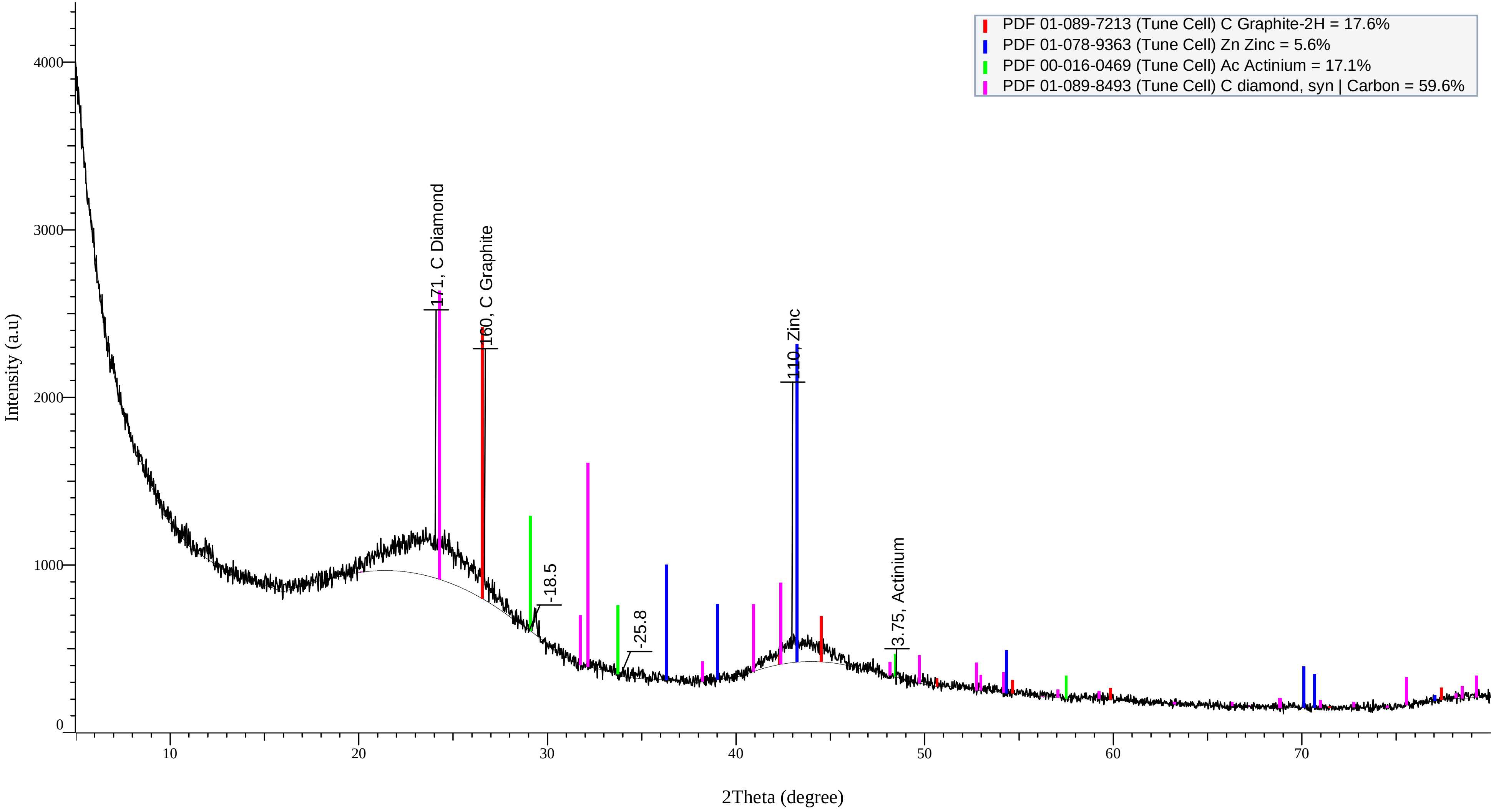

ZnAc2 - CAC Overall Data

**Visible**	Yes
**Icon**	
**Color**	
**Index**	1
**Name**	CAC- ZnAC2.raw
**Parent**	2Theta
**Sample Name**	CAC- ZnAC2
**Scan's Sample Name**	
**File Name**	CAC- ZnAC2.raw
**Experiment File Name**	
**Database ID**	
**Scan Type**	Coupled TwoTheta/Theta
**Scan Mode**	Continuousscan
**Scan Status**	Completed
**Start**	5.000
**End**	79.986
**Step Size**	0.025
**Time per Step**	19.00
**Axis Offset**	
**Temperature**	25 °C (Room)
**Temp. Diff.**	
**Heating Rate**	
**Time Started**	15.0
**Goniometer Radius**	217.5
**Goniometer Radius Primary**	
**Goniometer Radius Secondary**	
**2-theta**	5.000
**2-theta Osc. Amplitude**	0.000
**2-theta Osc. Speed**	0.000
**Theta**	2.500
**Theta Osc. Amplitude**	0.000
**Theta Osc. Speed**	0.000
**Detector**	
**Detector Osc. Amplitude**	
**Detector Osc. Speed**	
**Chi**	0.00
**Chi Osc. Amplitude**	0.00
**Chi Osc. Speed**	0.00
**Phi**	0.00
**Phi Osc. Amplitude**	0.00

**Phi Osc. Speed**	0.00
**Gamma**	0.00
**X-Drive**	0.0
**X-drive Osc. Amplitude**	0.0
**X-drive Osc. Speed**	0.0
**Y-Drive**	0.0
**Y-drive Osc. Amplitude**	0.0
**Y-drive Osc. Speed**	0.0
**Z-Drive**	0.0
**Z-drive Osc. Amplitude**	0.0
**Z-drive Osc. Speed**	0.0
**Aux1**	0.0
**Aux2**	0.0
**Aux3**	0.0
**Anode**	Cu
**kα1**	1.54060
**kα2**	1.54439
**kα2 Ratio**	0.50000
**kβ**	1.39222
**Generator kV**	40.0
**Generator mA**	40.0
**Detector Name**	Braun
**Calcium Channel**	
**Calcium Channel Time**	
**LynxEye 0D**	No
**PSD Opening**	
**PSD Opening Initial**	
**2Theta For Final PSD Opening**	
**Detector Slit Opening**	12.930
**Lower Discriminator**	
**Upper Discriminator**	
**Discriminator Width**	
**Detector Counts 0D**	
**Primary Soller slit**	4.000
**Secondary Soller slit**	4.000
**Center Soller slit**	
**Sample rotation speed**	
**Divergence Slit**	0.298
**Antiscatter Slit**	0.298
**Slit Mode**	Fixed
**Simul. Slit Mode**	
**Center Slit**	

**X-Offset**	0.000
**Displacement**	0.000
**Y-Scale Factor**	1
**Y-Offset**	0
**Humidity**	n.a.
**Curvature**	1.000
**Threshold**	1.000
**Enhanced**	No
**BackgroundFit**	No
**BackgroundOrder**	0.000
**BackgroundHyperbola**	No
**Compute Crystallinity**	Yes
**Cristallinity - From**	5.000
**Cristallinity - To**	79.986
**%-Crystallinity**	31.7%
**%-Amorphous**	68.3%
**Global Area**	343.0
**Reduced Area**	108.6
**Transmission without Blank**	
**Transmission**	
**Company Name**	Malaysia
**Operator Name**	Administrator
**User Comment**	
**Sample Position**	
**Creation Date/Time**	4/1/2017 3:52:01 PM
**Last Write Date/Time**	4/1/2017 3:52:01 PM
**Measurement Duration**	00:00:00
**Set index**	Not used

ZnAc2 - CAC Compute Crystallinity

**Index**	1
**Sample Name**	CAC- ZnAC2
**%-Crystallinity**	31.7%
**%-Amorphous**	68.3%
**Global Area**	343.0
**Reduced Area**	108.6

Modified KOH - CAC

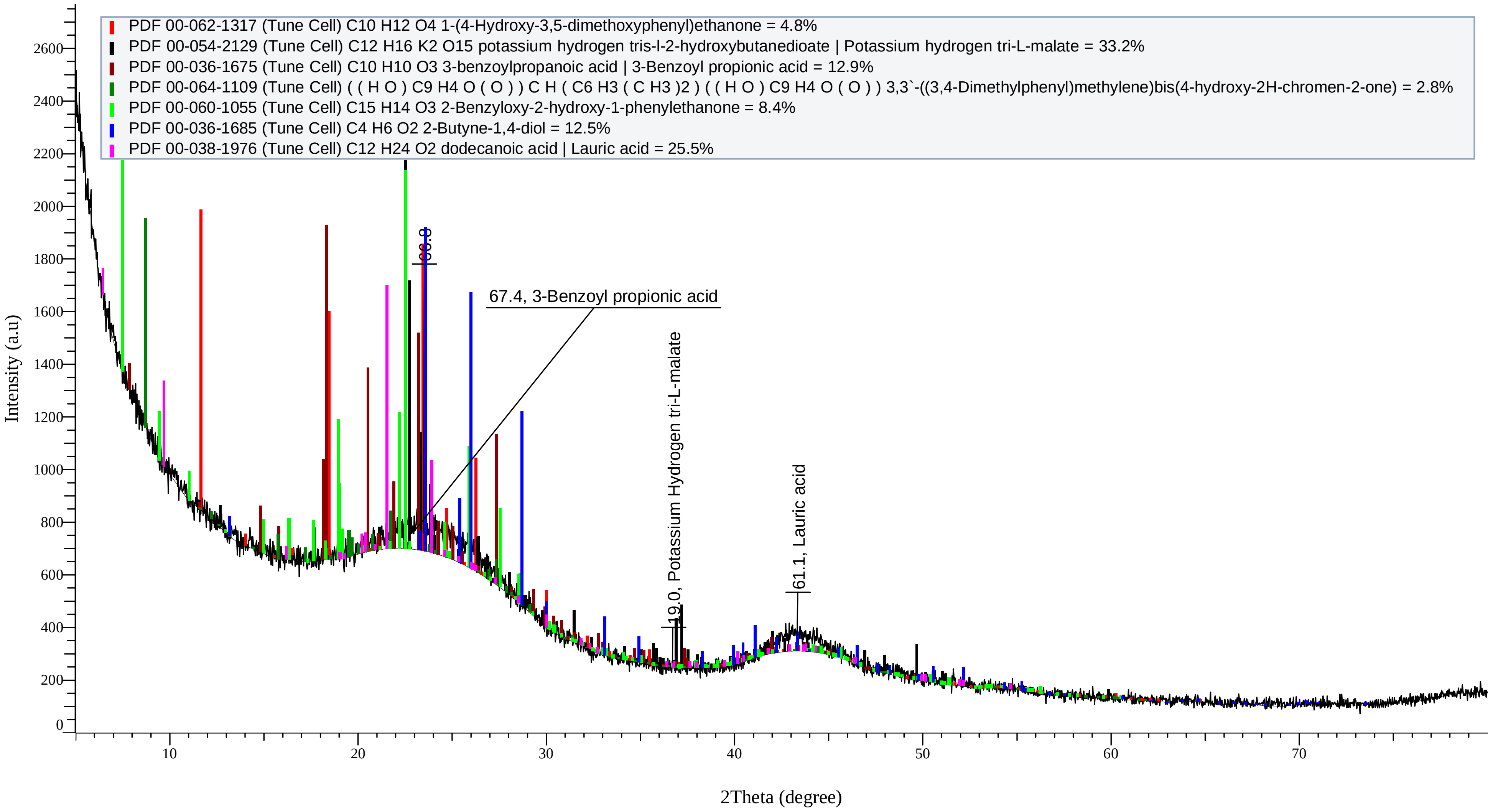

KOH - CAC Overall Data

**Visible**	Yes
**Icon**	
**Color**	
**Index**	1
**Name**	CAC- KOH.raw
**Parent**	2Theta
**Sample Name**	CAC- KOH
**Scan's Sample Name**	
**File Name**	CAC- KOH.raw
**Experiment File Name**	
**Database ID**	
**Scan Type**	Coupled TwoTheta/Theta
**Scan Mode**	Continuousscan
**Scan Status**	Completed
**Start**	5.000
**End**	79.986
**Step Size**	0.025
**Time per Step**	19.00
**Axis Offset**	
**Temperature**	25 °C (Room)
**Temp. Diff.**	
**Heating Rate**	
**Time Started**	15.0
**Goniometer Radius**	217.5
**Goniometer Radius Primary**	
**Goniometer Radius Secondary**	
**2-theta**	5.000
**2-theta Osc. Amplitude**	0.000
**2-theta Osc. Speed**	0.000
**Theta**	2.500
**Theta Osc. Amplitude**	0.000
**Theta Osc. Speed**	0.000
**Detector**	
**Detector Osc. Amplitude**	
**Detector Osc. Speed**	
**Chi**	0.00
**Chi Osc. Amplitude**	0.00
**Chi Osc. Speed**	0.00
**Phi**	0.00
**Phi Osc. Amplitude**	0.00

**Phi Osc. Speed**	0.00
**Gamma**	0.00
**X-Drive**	0.0
**X-drive Osc. Amplitude**	0.0
**X-drive Osc. Speed**	0.0
**Y-Drive**	0.0
**Y-drive Osc. Amplitude**	0.0
**Y-drive Osc. Speed**	0.0
**Z-Drive**	0.0
**Z-drive Osc. Amplitude**	0.0
**Z-drive Osc. Speed**	0.0
**Aux1**	0.0
**Aux2**	0.0
**Aux3**	0.0
**Anode**	Cu
**kα1**	1.54060
**kα2**	1.54439
**kα2 Ratio**	0.50000
**kβ**	1.39222
**Generator kV**	40.0
**Generator mA**	40.0
**Detector Name**	Braun
**Calcium Channel**	
**Calcium Channel Time**	
**LynxEye 0D**	No
**PSD Opening**	
**PSD Opening Initial**	
**2Theta For Final PSD Opening**	
**Detector Slit Opening**	12.930
**Lower Discriminator**	
**Upper Discriminator**	
**Discriminator Width**	
**Detector Counts 0D**	
**Primary Soller slit**	4.000
**Secondary Soller slit**	4.000
**Center Soller slit**	
**Sample rotation speed**	
**Divergence Slit**	0.298
**Antiscatter Slit**	0.298
**Slit Mode**	Fixed
**Simul. Slit Mode**	
**Center Slit**	

**X-Offset**	0.000
**Displacement**	0.000
**Y-Scale Factor**	1
**Y-Offset**	0
**Humidity**	n.a.
**Curvature**	1.000
**Threshold**	1.000
**Enhanced**	No
**BackgroundFit**	No
**BackgroundOrder**	0.000
**BackgroundHyperbola**	No
**Compute Crystallinity**	Yes
**Cristallinity - From**	5.000
**Cristallinity - To**	79.986
**%-Crystallinity**	31.3%
**%-Amorphous**	68.7%
**Global Area**	194.2
**Reduced Area**	60.82
**Transmission without Blank**	
**Transmission**	
**Company Name**	Malaysia
**Operator Name**	Administrator
**User Comment**	
**Sample Position**	
**Creation Date/Time**	4/1/2017 4:03:20 PM
**Last Write Date/Time**	4/1/2017 4:03:20 PM
**Measurement Duration**	00:00:00
**Set index**	Not used

KOH - CAC Compute Crystallinity

**Index**	1
**Sample Name**	CAC- KOH
**%-Crystallinity**	31.3%
**%-Amorphous**	68.7%
**Global Area**	194.2
**Reduced Area**	60.82

Modified KI - CAC

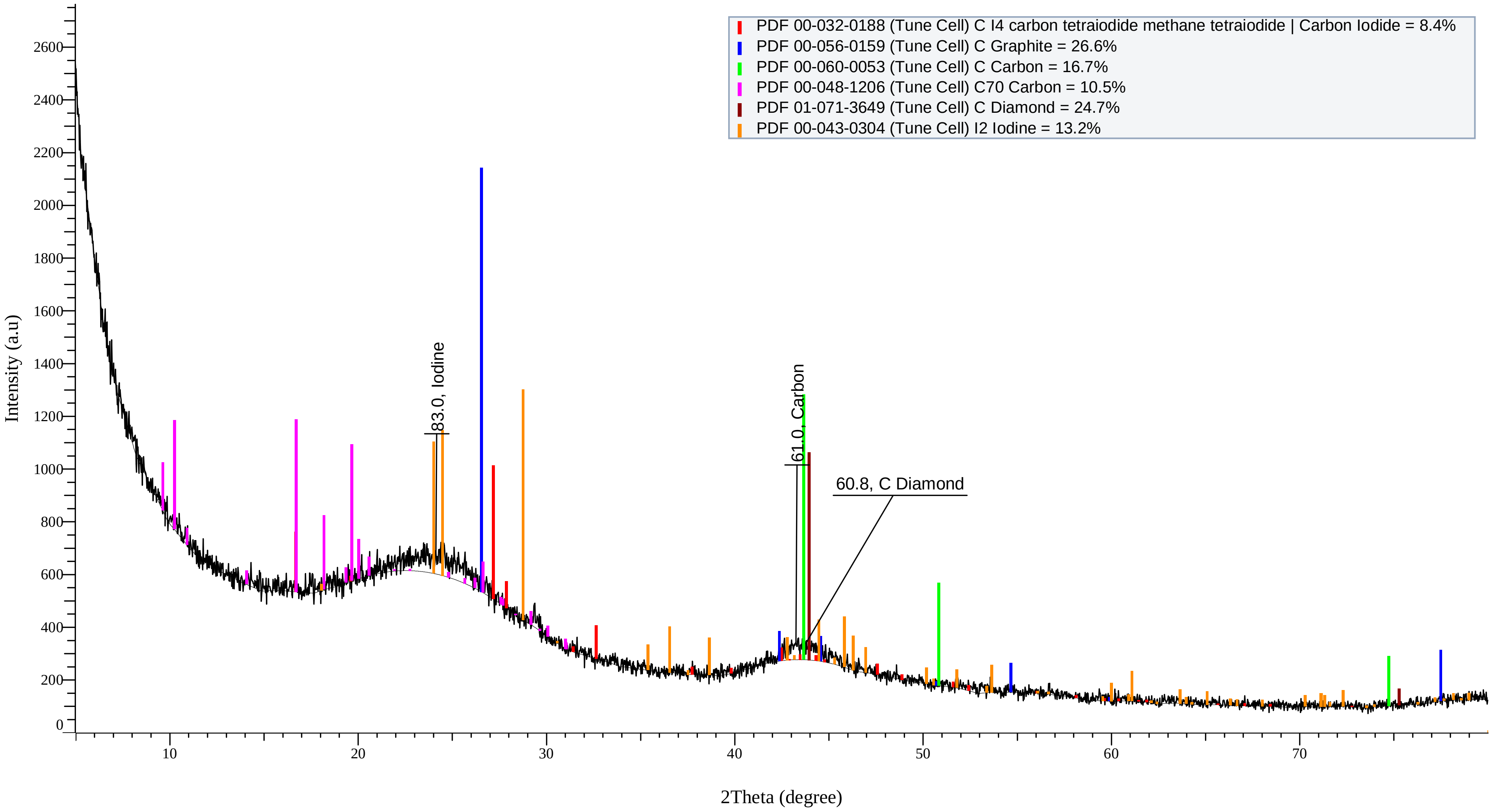

KI - CAC Overall Data

Visible	Yes
Icon	
Color	
Index	1
Name	CAC- KI.raw
Parent	2Theta
Sample Name	CAC- KI
Scan's Sample Name	
File Name	CAC- KI.raw
Experiment File Name	
Database ID	
Scan Type	Coupled TwoTheta/Theta
Scan Mode	Continuousscan
Scan Status	Completed
Start	5.000
End	79.986
Step Size	0.025
Time per Step	19.00
Axis Offset	
Temperature	25 °C (Room)
Temp. Diff.	
Heating Rate	
Time Started	14.0
Goniometer Radius	217.5
Goniometer Radius Primary	
Goniometer Radius Secondary	
2-theta	5.000
2-theta Osc. Amplitude	0.000
2-theta Osc. Speed	0.000
Theta	2.500
Theta Osc. Amplitude	0.000
Theta Osc. Speed	0.000
Detector	
Detector Osc. Amplitude	
Detector Osc. Speed	
Chi	0.00
Chi Osc. Amplitude	0.00
Chi Osc. Speed	0.00
Phi	0.00
Phi Osc. Amplitude	0.00

**Phi Osc. Speed**	0.00
**Gamma**	0.00
**X-Drive**	0.0
**X-drive Osc. Amplitude**	0.0
**X-drive Osc. Speed**	0.0
**Y-Drive**	0.0
**Y-drive Osc. Amplitude**	0.0
**Y-drive Osc. Speed**	0.0
**Z-Drive**	0.0
**Z-drive Osc. Amplitude**	0.0
**Z-drive Osc. Speed**	0.0
**Aux1**	0.0
**Aux2**	0.0
**Aux3**	0.0
**Anode**	Cu
**kα1**	1.54060
**kα2**	1.54439
**kα2 Ratio**	0.50000
**kβ**	1.39222
**Generator kV**	40.0
**Generator mA**	40.0
**Detector Name**	Braun
**Calcium Channel**	
**Calcium Channel Time**	
**LynxEye 0D**	No
**PSD Opening**	
**PSD Opening Initial**	
**2Theta For Final PSD Opening**	
**Detector Slit Opening**	12.930
**Lower Discriminator**	
**Upper Discriminator**	
**Discriminator Width**	
**Detector Counts 0D**	
**Primary Soller slit**	4.000
**Secondary Soller slit**	4.000
**Center Soller slit**	
**Sample rotation speed**	
**Divergence Slit**	0.298
**Antiscatter Slit**	0.298
**Slit Mode**	Fixed
**Simul. Slit Mode**	
**Center Slit**	

**X-Offset**	0.000
**Displacement**	0.000
**Y-Scale Factor**	1
**Y-Offset**	0
**Humidity**	n.a.
**Curvature**	1.000
**Threshold**	1.000
**Enhanced**	No
**BackgroundFit**	No
**BackgroundOrder**	0.000
**BackgroundHyperbola**	No
**Compute Crystallinity**	Yes
**Cristallinity - From**	5.000
**Cristallinity - To**	79.986
**%-Crystallinity**	26.4%
**%-Amorphous**	73.6%
**Global Area**	187.5
**Reduced Area**	49.59
**Transmission without Blank**	
**Transmission**	
**Company Name**	Malaysia
**Operator Name**	Administrator
**User Comment**	
**Sample Position**	
**Creation Date/Time**	4/1/2017 3:57:40 PM
**Last Write Date/Time**	4/1/2017 3:57:40 PM
**Measurement Duration**	00:00:00
**Set index**	Not used

KI - CAC Compute Crystallinity

**Index**	1
**Sample Name**	CAC- KI
**%-Crystallinity**	26.4%
**%-Amorphous**	73.6%
**Global Area**	187.5
**Reduced Area**	49.59

Modified CuSO4 - CAC

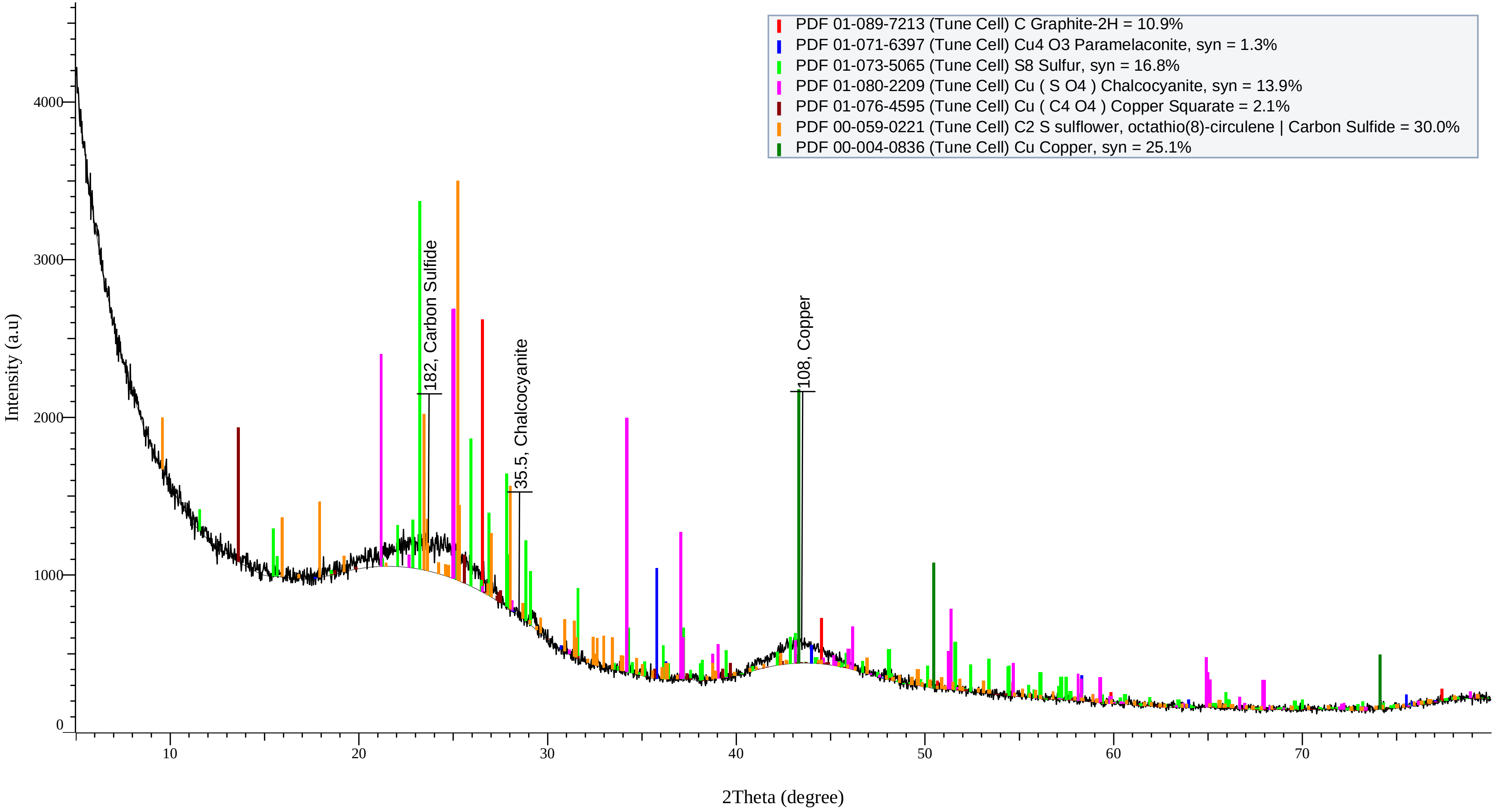

CuSO4- CAC Overall Data

**Visible**	Yes
**Icon**	
**Color**	
**Index**	1
**Name**	CAC- CuSO4.raw
**Parent**	2Theta
**Sample Name**	CAC- CuSO4
**Scan's Sample Name**	
**File Name**	CAC- CuSO4.raw
**Experiment File Name**	
**Database ID**	
**Scan Type**	Coupled TwoTheta/Theta
**Scan Mode**	Continuousscan
**Scan Status**	Completed
**Start**	5.000
**End**	79.986
**Step Size**	0.025
**Time per Step**	19.00
**Axis Offset**	
**Temperature**	25 °C (Room)
**Temp. Diff.**	
**Heating Rate**	
**Time Started**	15.0
**Goniometer Radius**	217.5
**Goniometer Radius Primary**	
**Goniometer Radius Secondary**	
**2-theta**	5.000
**2-theta Osc. Amplitude**	0.000
**2-theta Osc. Speed**	0.000
**Theta**	2.500
**Theta Osc. Amplitude**	0.000
**Theta Osc. Speed**	0.000
**Detector**	
**Detector Osc. Amplitude**	
**Detector Osc. Speed**	
**Chi**	0.00
**Chi Osc. Amplitude**	0.00
**Chi Osc. Speed**	0.00
**Phi**	0.00
**Phi Osc. Amplitude**	0.00

**Phi Osc. Speed**	0.00
**Gamma**	0.00
**X-Drive**	0.0
**X-drive Osc. Amplitude**	0.0
**X-drive Osc. Speed**	0.0
**Y-Drive**	0.0
**Y-drive Osc. Amplitude**	0.0
**Y-drive Osc. Speed**	0.0
**Z-Drive**	0.0
**Z-drive Osc. Amplitude**	0.0
**Z-drive Osc. Speed**	0.0
**Aux1**	0.0
**Aux2**	0.0
**Aux3**	0.0
**Anode**	Cu
**kα1**	1.54060
**kα2**	1.54439
**kα2 Ratio**	0.50000
**kβ**	1.39222
**Generator kV**	40.0
**Generator mA**	40.0
**Detector Name**	Braun
**Calcium Channel**	
**Calcium Channel Time**	
**LynxEye 0D**	No
**PSD Opening**	
**PSD Opening Initial**	
**2Theta For Final PSD Opening**	
**Detector Slit Opening**	12.930
**Lower Discriminator**	
**Upper Discriminator**	
**Discriminator Width**	
**Detector Counts 0D**	
**Primary Soller slit**	4.000
**Secondary Soller slit**	4.000
**Center Soller slit**	
**Sample rotation speed**	
**Divergence Slit**	0.298
**Antiscatter Slit**	0.298
**Slit Mode**	Fixed
**Simul. Slit Mode**	
**Center Slit**	

**X-Offset**	0.000
**Displacement**	0.000
**Y-Scale Factor**	1
**Y-Offset**	0
**Humidity**	n.a.
**Curvature**	1.000
**Threshold**	1.000
**Enhanced**	No
**BackgroundFit**	No
**BackgroundOrder**	0.000
**BackgroundHyperbola**	No
**Compute Crystallinity**	Yes
**Cristallinity - From**	5.000
**Cristallinity - To**	79.986
**%-Crystallinity**	31.6%
**%-Amorphous**	68.4%
**Global Area**	329.1
**Reduced Area**	104.0
**Transmission without Blank**	
**Transmission**	
**Company Name**	Malaysia
**Operator Name**	Administrator
**User Comment**	
**Sample Position**	
**Creation Date/Time**	4/1/2017 3:46:21 PM
**Last Write Date/Time**	4/1/2017 3:46:21 PM
**Measurement Duration**	00:00:00
**Set index**	Not used

CuSO4 - CAC Compute Crystallinity

**Index**	1
**Sample Name**	CAC- CuSO4
**%-Crystallinity**	31.6%
**%-Amorphous**	68.4%
**Global Area**	329.1
**Reduced Area**	104.0
