# Supplementary material for: Removal of hydrogen sulfide from a biogas mimic by using impregnated activated carbon adsorbent
Source: PLoS One. 2019 Feb 12;14(2):e0211713. doi: 10.1371/journal.pone.0211713 (PMC6372171; doi:10.1371/journal.pone.0211713)
Supplement: S4 Table — (PDF) [file pone.0211713.s005.pdf]

Sample:	001-783-ZnAC2_CAC_Adsorp
Operator:	Jack
Submitter:	Amy
Bar Code:	
File:	C:\2020\data_ini\001-783.SMP

Started:	04-Nov-17 7:31:28 AM	Analysis adsorptive:	N2
Completed:	04-Nov-17 5:49:57 PM	Analysis bath temp.:	-195.751 °C
Report time:	13-Nov-17 6:31:54 PM	Thermal correction:	No
Sample mass:	0.1680 g	Warm free space:	27.7257 cm³ Measured
Cold free space:	85.2131 cm³	Equilibration interval:	10 s
Low pressure dose:	5.0000 cm³/g STP	Sample density:	1.000 g/cm³
Automatic degas:	Yes		

Summary Report

Surface Area

Single point surface area at P/Po = 0.201206492:	643.0161 m²/g
BET Surface Area:	625.4326 m²/g
Langmuir Surface Area:	826.5136 m²/g
t-Plot Micropore Area:	512.1831 m²/g
t-Plot external surface area:	113.2495 m²/g
BJH Adsorption cumulative surface area of pores between 17.000 Å and 3,000.000 Å width:	48.988 m²/g

BJH Desorption cumulative surface area of pores

between 17.000 Å and 3,000.000 Å width:	52.8711 m^2/g

Pore Volume

Single point adsorption total pore volume of pores less than 1,294.169 Å width at P/Po = 0.984813395:	0.301218 cm^3/g
Single point desorption total pore volume of pores less than 755.975 Å width at P/Po = 0.973713272:	0.300687 cm^3/g
t-Plot micropore volume:	0.236326 cm^3/g
BJH Adsorption cumulative volume of pores between 17.000 Å and 3,000.000 Å width:	0.040593 cm^3/g
BJH Desorption cumulative volume of pores between 17.000 Å and 3,000.000 Å width:	0.041372 cm^3/g

Pore Size

Adsorption average pore diameter (4V/A by BET):	19.2646 Å
Desorption average pore diameter (4V/A by BET):	19.2306 Å
BJH Adsorption average pore width (4V/A):	33.145 Å
BJH Desorption average pore width (4V/A):	31.300 Å

Sample:	001-782-ZnAC2-CAC-Desorp
Operator:	Jack
Submitter:	Amy
Bar Code:	
File:	C:\2020\data_ini\001-782.SMP

Started:	03-Nov-17 9:41:47 AM	Analysis adsorptive:	N2
Completed:	03-Nov-17 10:08:38 PM	Analysis bath temp.:	-195.771 °C
Report time:	13-Nov-17 6:31:53 PM	Thermal correction:	No
Sample mass:	0.1767 g	Warm free space:	27.5616 cm³ Measured
Cold free space:	85.0576 cm³	Equilibration interval:	10 s
Low pressure dose:	5.0000 cm³/g STP	Sample density:	1.000 g/cm³
Automatic degas:	Yes		

Summary Report

Surface Area

Single point surface area at P/Po = 0.201178113:	740.0198 m²/g
BET Surface Area:	717.4079 m²/g
Langmuir Surface Area:	953.2622 m²/g
t-Plot Micropore Area:	578.5584 m²/g
t-Plot external surface area:	138.8495 m²/g
BJH Adsorption cumulative surface area of pores between 17.000 Å and 3,000.000 Å width:	60.398 m²/g

BJH Desorption cumulative surface area of pores

between 17.000 Å and 3,000.000 Å width:	65.5456 m^2/g

Pore Volume

Single point adsorption total pore volume of pores less than 1,308.001 Å width at P/Po = 0.984976849:	0.348118 cm^3/g
Single point desorption total pore volume of pores less than 754.765 Å width at P/Po = 0.973670152:	0.347410 cm^3/g
t-Plot micropore volume:	0.268258 cm^3/g
BJH Adsorption cumulative volume of pores between 17.000 Å and 3,000.000 Å width:	0.048945 cm^3/g
BJH Desorption cumulative volume of pores between 17.000 Å and 3,000.000 Å width:	0.050661 cm^3/g

Pore Size

Adsorption average pore diameter (4V/A by BET):	19.4098 Å
Desorption average pore diameter (4V/A by BET):	19.3703 Å
BJH Adsorption average pore width (4V/A):	32.415 Å
BJH Desorption average pore width (4V/A):	30.917 Å
